# Combined emission ratiometry and motion tracking for optical mapping of contracting hearts: Validation with monophasic action potentials

**DOI:** 10.1113/JP291400

**Published:** 2026-06-16

**Authors:** Vineesh Kappadan, Zhen Hua, Johanna B. Tonko, Najmah Mohamed, Jan Lebert, Paraskevas Efstathiou, Yasser Abdelghani, Danya Agha‐Jaffar, Anies Sohi, Xianbo Sun, Jean‐Baptiste Guichard, Jan Christoph, Nicholas S. Peters, Fu Siong Ng

**Affiliations:** ^1^ National Heart and Lung Institute (NHLI) Imperial College London London UK; ^2^ Queen Mary University of London London UK; ^3^ Cardiovascular Research Institute University of California San Francisco CA USA; ^4^ Institut Clínic Cardiovascular (ICCV) Hospital Clínic, Universitat de Barcelona Barcelona Catalonia Spain; ^5^ Institut d'Investigacions Biomèdiques August Pi i Sunyer (IDIBAPS) Barcelona Catalonia Spain

**Keywords:** contracting heart, emission ratiometry, monophasic action potentials, motion artefact, motion tracking, optical mapping

## Abstract

**Abstract:**

Cardiac optical mapping in fully contracting hearts is limited by motion artefacts, leading most investigations to rely on excitation–contraction uncoupling despite its impact on physiological relevance. Although motion tracking and ratiometric imaging have each been used to reduce motion artefacts, and their combined use has been demonstrated with excitation ratiometry, systematic validation of emission ratiometry combined with motion tracking against an established electrophysiological reference remains limited.

In this study, we integrated emission ratiometry with two‐dimensional, marker‐free motion tracking to suppress motion‐related artefacts during optical mapping of Langendorff‐perfused rabbit hearts, leveraging the advantage that emission ratiometry derives both signals from the same excitation pathway and thereby reduces sensitivity to motion‐related photometric fluctuations in fluorescence intensity. Motion‐corrected optical action potentials were quantitatively validated against simultaneously recorded monophasic action potentials (MAPs), with optical signals extracted from regions adjacent to the MAP electrode. Compared with raw recordings and with either method applied alone, the combined approach produced the closest agreement with MAP‐derived repolarization measurements. Bland–Altman analysis indicated no systematic bias at APD_70_ and APD_80_, with bias confidence intervals including zero. Cumulative probability analysis revealed a marked improvement in precision following combined motion correction, with the proportion of optical action potential duration (APD) measurements within ±10 ms of MAP increasing from 16.9%, 29.2% and 26.2% in raw recordings to 75.4%, 87.7% and 80% for APD_50_, APD_70_ and APD_80_, respectively.

Collectively, these findings establish the integrated motion‐correction strategy as a validated framework for accurate quantification of action potentials in contracting hearts, supporting more physiologically relevant optical mapping.

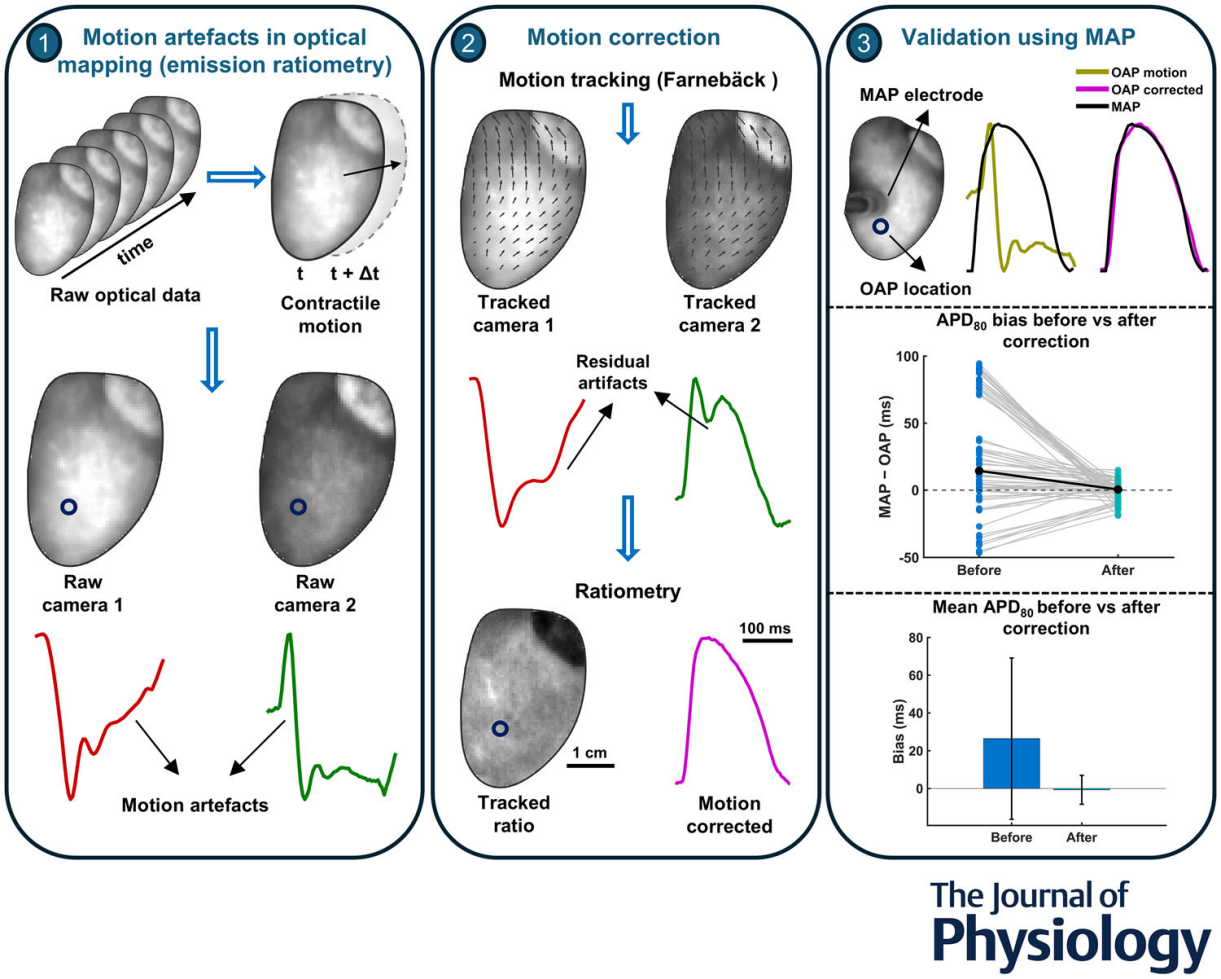

**New & noteworthy:**

While motion tracking and ratiometric approaches have previously been used to mitigate motion artefacts in optical mapping, this study is the first to integrate emission ratiometry with fully marker‐free motion tracking and to provide systematic validation against simultaneously recorded monophasic action potentials in freely contracting isolated rabbit hearts. This validated framework enables accurate optical assessment of cardiac electrical activity under physiologically relevant conditions without mechanical stabilization or excitation–contraction uncoupling.

## Graphical abstract

**Abstract figure legend** Motion‐correction pipeline and validation with monophasic action potential (MAP). *Left*, contractile motion introduces artefacts in dual‐camera optical signals, with dual‐emission imaging used for ratiometry. *Middle*, motion correction using motion tracking and ratiometry, with displacement vectors overlaid on images and improved signals after motion tracking and after tracking with ratiometry. *Right*, validation against MAP shows distorted optical action potentials before correction and good agreement after correction, with reduced APD_80_ bias and reduced mean APD_80_ bias.

## Introduction

Cardiac optical mapping is a powerful imaging technique that enables high‐resolution visualization of cardiac electrophysiological activity using voltage‐ and calcium‐sensitive fluorescent dyes (Attin & Clusin, 2009; Efimov et al., 2004; Salama & Morad, 1976). This approach permits simultaneous measurement of transmembrane action potentials and intracellular calcium dynamics with excellent spatial and temporal resolution (Choi & Salama, 2000; Herron et al., 2012; Lee et al., 2012). Advances in instrumentation have enabled multi‐parametric configurations incorporating metabolic signals such as reduced nicotinamide adenine dinucleotide (NADH) and flavin adenine dinucleotide (FAD) (George et al., 2022; Lee et al., 2012; Mills & George, 2025; Swift et al., 2008), further expanding its utility. Optical mapping has therefore played a central role in advancing the understanding of cardiac excitation, conduction, repolarization heterogeneity and arrhythmogenesis in isolated heart and tissue preparations (Efimov et al., 1994, 2004; Handa et al., 2020; Lou et al., 2012; Renard et al., 2024; Wang et al., 2015).

A major limitation of optical mapping arises from motion artefacts associated with cardiac contraction (Christoph & Luther, 2018; Kappadan et al., 2023; Svrcek et al., 2009). Pharmacological excitation–contraction (EC) uncoupling, most commonly using blebbistatin, suppresses mechanical activity while largely preserving electrical excitation and has facilitated widespread adoption of the technique (Fedorov et al., 2007; Swift et al., 2021). However, eliminating contraction also alters myocardial physiology in fundamental ways. Mechanical arrest disrupts EC coupling and abolishes mechano‐electric feedback, a bidirectional mechanism through which myocardial deformation modulates electrical activity and contributes to normal cardiac function and arrhythmogenesis (Franz, 1996, 2000; Franz & Bode, 2003; Kohl & Ravens, 2003). It also substantially reduces myocardial metabolic demand and oxygen consumption (Kuzmiak‐Glancy *et al.*, 2015). Together, these effects, alongside reported electrophysiological alterations in both cellular and whole‐heart preparations under EC‐uncoupled conditions (Baker et al., 2004; Brack et al., 2013; Kappadan et al., 2020; Lou et al., 2012; Sobitov et al., 2026; Swift et al., 2021), raise the possibility that electrophysiological measurements obtained under EC‐uncoupled conditions differ from those observed in actively contracting myocardium, underscoring the importance of developing optical mapping approaches that preserve cardiac contraction.

Recent advances in computational motion tracking and ratiometric imaging have enabled optical mapping of electrophysiological activity in isolated contracting hearts without pharmacological EC uncoupling (Garrott et al., 2017; Kappadan et al., 2023; Nesmith et al., 2020). In contracting preparations, motion tracking reduces artefacts arising from a loss of spatial correspondence between myocardial tissue and the imaging sensor during the cardiac cycle. Motion tracking approaches can be broadly categorized as marker‐based (Zhang et al., 2016, 2023) or marker‐free (Christoph & Luther, 2018; Christoph et al., 2017). Marker‐based methods estimate tissue displacement by tracking fiducial markers attached to the epicardial surface, increasing experimental complexity through the need for physical attachment and often requiring additional imaging views or geometric calibration. Since motion information is obtained only at discrete marker locations, reconstruction of a continuous deformation field requires interpolation between markers, and the effective spatial resolution of motion estimates depends largely on marker density. In contrast, marker‐free approaches based on optical flow, such as the Farnebäck method (Lebert et al., 2022), estimate displacement directly from intrinsic image intensity patterns across successive frames, enabling motion estimation across the entire imaged surface without physical markers. However, motion tracking primarily compensates for geometric displacement between the tissue and the imaging sensor and therefore does not fully eliminate residual photometric artefacts in optical action potentials (OAPs) recorded from contracting hearts. These residual fluctuations arise in part from apparent movement of illumination across the tissues, as well as from factors such as non‐uniform illumination, variable viewing geometry, heterogeneous dye loading, photobleaching and dye internalization. Ratiometric optical mapping provides a complementary strategy by attenuating photometric fluctuations shared across paired fluorescence measurements. In excitation ratiometry, a voltage‐sensitive dye is alternately excited at two wavelengths while fluorescence is collected within a single emission band (Bachtel et al., 2011; Chowdhary et al., 2026; Kappadan et al., 2020; Lee et al., 2019; Zhang et al., 2016). Although effective, this approach acquires the two signals on successive frames, so contraction can introduce temporal mismatch between channels, while wavelength‐dependent differences in absorption and scattering may further limit artefact cancellation. In contrast, emission ratiometry uses a single excitation wavelength while simultaneously recording fluorescence in two spectrally distinct emission bands, allowing shared photometric artefacts to be matched in time and more effectively attenuated, provided the two detection channels are spatially aligned accurately (Knisley et al., 2000; Kong et al., 2003).

In earlier work, we validated the approach using synthetic optical mapping datasets with known ground truth (Christoph & Luther, 2018). We subsequently evaluated marker‐free motion tracking in contracting hearts by comparing optical signals with microelectrode recordings (Kappadan et al., 2023). We further combined marker‐free motion tracking with emission ratiometry to investigate electrophysiological changes during regional ischaemia in contracting hearts (Kappadan et al., 2026). However, the approach was not subjected to systematic quantitative validation. Although validation against monophasic action potentials (MAPs) has previously been reported using marker‐based tracking combined with excitation ratiometry (Zhang et al., 2023), an equivalent validation for marker‐free motion tracking with emission ratiometry remains lacking. In the present study, we address this gap by combining two‐dimensional (2D) marker‐free motion tracking with emission ratiometry during optical mapping of freely contracting Langendorff‐perfused rabbit hearts. Motion‐corrected OAPs are quantitatively validated against simultaneously recorded MAPs, enabling rigorous assessment of action potential repolarization timing across multiple repolarization levels under physiologically relevant conditions that preserve EC coupling and mechano‐electric feedback, albeit without the full physiological preload and afterload conditions of pressure‐volume (PV) working heart preparations.

## Methods

### Experimental preparation

A total of seven female New Zealand White rabbits (10–12 weeks old, 2–2.5 kg) were obtained from Inotiv, UK. Animals were sedated with Domitor (1 mg/mL, 0.125 mL/kg; Orion Pharma, UK) in combination with Ketavet (100 mg/mL, 0.075 mL/kg; Zoetis, UK). After approximately 15 min, the rabbits were killed by administering an overdose of anaesthesia (pentobarbitone sodium, 200 mg/mL, 0.8 mL/kg; Animal Care, UK) intravenously through the marginal ear vein. To prevent blood coagulation, 2500 IU of heparin (Panpharma, UK) was administered concurrently.

After thoracotomy, hearts were promptly excised, cannulated via the aorta and perfused retrogradely using a Langendorff perfusion system. The perfusate consisted of oxygenated (95% O_2_ and 5% CO_2_) Tyrode's solution containing (in mmol/L): NaCl 128.2, CaCl_2_ 1.3, KCl 4.7, MgCl_2_ 1.05, NaH_2_PO_4_ 1.19, NaHCO_3_ 20 and glucose 11.1. Hearts were maintained at 37°C in a temperature‐controlled acrylic chamber and perfused at a constant flow rate of 30 mL/min. Electrocardiogram (ECG) signals were continuously monitored via needle electrodes connected to a Bio Amp system and a PowerLab 8/30 data acquisition unit (both from ADInstruments, Australia). For pacing, a bipolar electrode was positioned on the right ventricle and connected to a Micropace III stimulator (MicropaceEP Ltd, California, USA) to deliver electrical stimulation.

All animal procedures were carried out in accordance with the UK Animals (Scientific Procedures) Act 1986 and approved by the institutional animal ethics committee.

### Optical mapping and monophasic action potential recordings

**Figure A1:**
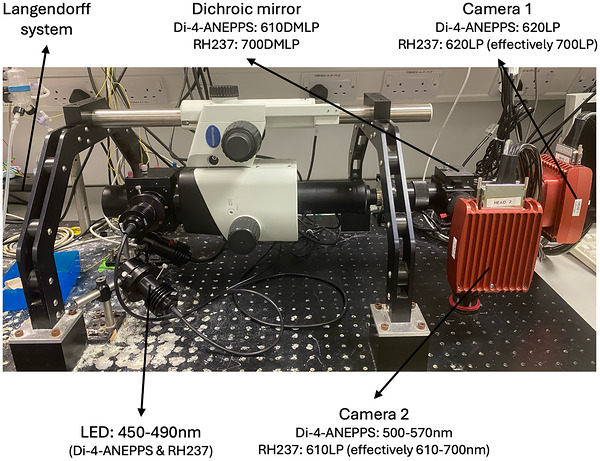
Experimental setup for ratiometric optical mapping of cardiac action potentials A Langendorff‐perfused heart is illuminated by blue LEDs (450‐490 nm excitation) used to excite both voltage‐sensitive dyes, Di‐4‐ANEPPS and RH237. Emitted fluorescence is split by a dichroic mirror (610DMLP for Di‐4‐ANEPPS; 700DMLP for RH237) and directed to two orthogonally oriented CMOS cameras (128 × 80 pixels, 250 Hz) aligned to image the same region of the epicardium. Camera 1 is fitted with a 620LP filter (effectively 700LP for RH237), capturing the long‐wavelength emission channel (red for Di‐4‐ANEPPS; near‐infrared for RH237). Camera 2 is fitted with a 535 ± 35 nm filter for Di‐4‐ANEPPS (green channel) or a 610LP filter (effectively 610–700 nm) for RH237 (red channel), capturing the short‐wavelength emission. This dual‐camera configuration enables simultaneous acquisition of the two spectral components required for ratiometric imaging of transmembrane potential, reducing motion and illumination artefacts.

Ratiometric optical mapping of cardiac action potentials was performed using the voltage‐sensitive dyes Di‐4‐ANEPPS (3–4 µM, VWR International Ltd., UK) and RH237 (2–5 µM, VWR International Ltd., UK), with fluorescence signals acquired at 250 Hz. Illumination was provided by four blue light‐emitting diodes (LEDs; 470 ± 20 nm, Cairn Research, UK) equipped with excitation filters of 450–490 nm. Fluorescence emission was recorded using two orthogonally oriented CMOS cameras (128 × 80 pixels) aligned to image the same region of the heart. Camera alignment was achieved using a two‐step procedure. First, a projected point light source was introduced into the optical system for gross mechanical co‐alignment. The camera fitted with the >620 nm filter was held fixed as the reference, while the second camera was adjusted through purely rigid‐body transformations until the projected light source appeared at the same position in both fields of view. Second, computational verification of the registration was performed by confirming the correspondence of shared anatomical landmarks visible in both channels, such as epicardial vessels, ensuring that matched pixels represented the same tissue location. The cameras were fitted with green (535 ± 35 nm) and red (>620 nm) emission filters (Chroma Technology, USA) to enable ratiometric imaging with Di‐4‐ANEPPS. For dual‐emission ratiometry of RH237, red (610–700 nm) and near‐infrared (>700 nm) emission filters (Chroma Technology, USA) were used. The experimental optical mapping setup is illustrated in Fig. A1. Monophasic action potential (MAP) signals were obtained using an MAP electrode, a Bio Amp system, and a PowerLab 8/30 data acquisition unit (all from ADInstruments, Australia). Optical mapping was performed with the MAP electrode positioned within the optical field of view and in direct contact with the ventricular surface, allowing simultaneous optical and MAP recordings.

Recordings were acquired under multiple electrophysiological conditions, including sinus rhythm, ventricular pacing at a cycle length of 280 ms, and ventricular fibrillation induced by rapid burst pacing (25 Hz; 40 ms cycle length). Quantitative comparisons between optical and MAP signals were restricted to sinus rhythm recordings because stable MAP electrode contact during pacing could not be consistently maintained across preparations, likely due to pacing‐induced changes in local mechanical motion and limitations imposed by the electrode design and the curvature of the epicardial surface. In addition, the spatiotemporal variability of ventricular fibrillation (VF), together with the slight spatial mismatch between the MAP electrode location and nearby optical recording sites precluded direct beat‐to‐beat comparisons during VF.

### Processing of optical mapping and monophasic action potential data

Optical mapping data were analysed using custom MATLAB and Python scripts. Prior to analysis of OAP signals, motion artefacts arising from cardiac contraction were minimized using a motion‐correction strategy that combined marker‐free motion tracking with emission ratiometry.

### Marker‐free motion tracking

2D marker‐free motion tracking was conducted using the *Farnebäck* optical flow algorithm (Lebert & Christoph, 2023) via the *optimap* library (https://github.com/cardiacvision/optimap/). To enable reliable motion tracking, the raw optical mapping video acquired from camera 1 was contrast‐enhanced using a kernel size of 5. This preprocessing step facilitates optical flow‐based motion estimation by reducing non‐motion‐related intensity fluctuations and improves compliance with the brightness constancy assumption. The use of contrast enhancement for marker‐free motion tracking in contracting, fluorescing hearts was motivated by our prior work that demonstrates numerical enhancement of image contrast substantially improves tracking robustness under such conditions. Cardiac motion was estimated by computing the geometrical transformation between a reference image and each subsequent test image. For data acquired during paced rhythm or sinus rhythm, the reference image was selected as the frame immediately preceding depolarization. For VF recordings, in which no consistent activation phase is present, an arbitrary frame within the sequence was chosen as the reference. The resulting displacement fields were applied uniformly to the original optical videos from both camera 1 and camera 2 to ensure consistent spatial deformation across datasets.

### Emission ratiometry of motion‐tracked videos

Ratiometric processing of motion‐tracked optical mapping videos suppresses shared photometric fluctuations associated with illumination heterogeneity, dye variability and other motion‐dependent optical factors. Because of a voltage‐dependent spectral shift of the dye emission spectrum during the action potential, fluorescence intensity decreases in the detection band of camera 1 (negative ΔF) and increases in the detection band of camera 2 (positive ΔF). Therefore, the ratio of the motion‐tracked camera 2 signal to the motion‐tracked camera 1 signal (camera 2/camera 1) produces a positive deflecting ratio signal during the action potential, which was used for subsequent analysis.

**Figure 1:**
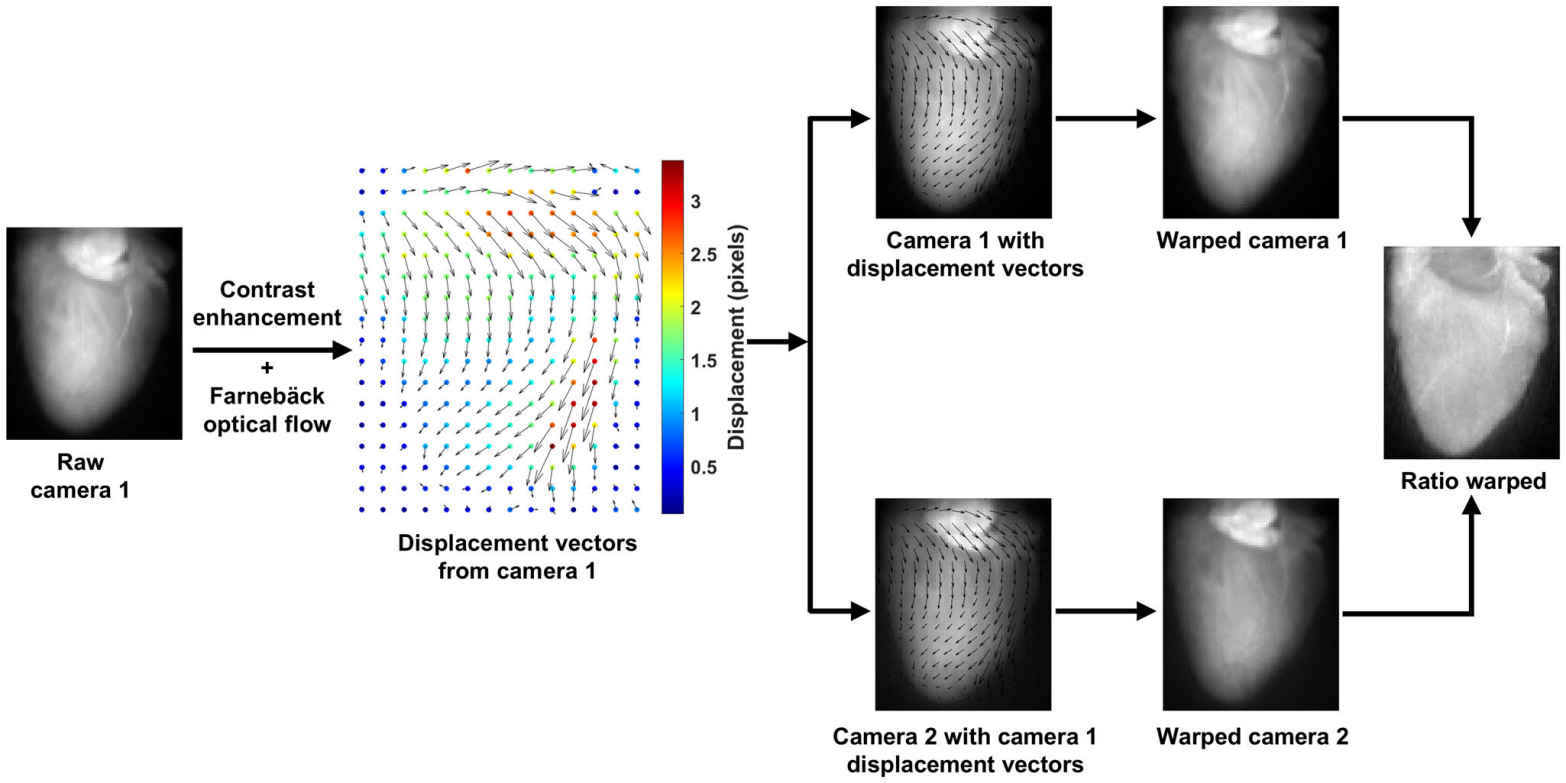
Motion‐correction pipeline for emission ratiometric optical mapping Tissue motion was estimated from the optical mapping video acquired by camera 1 by computing displacement vectors using the Farnebäck optical flow method. These displacement vectors were then applied to warp the videos from both cameras, yielding motion‐tracked videos. Ratioing the motion‐tracked videos attenuates shared photometric variations across channels.

Fig. 1 illustrates the motion‐correction pipeline used in this study. The raw optical mapping video from camera 1 was first contrast‐enhanced to facilitate motion estimation. Motion tracking was then performed on the enhanced video using the Farnebäck optical flow method to compute displacement vector fields relative to a reference frame. These displacement fields, derived from camera 1, were applied to both the original camera 1 and camera 2 videos to ensure identical spatial warping across both emission channels, which is required for accurate emission ratiometry. Following spatial warping, emission ratiometry was performed to reduce shared photometric fluctuations.

Together, this combined motion tracking and ratiometry framework enables robust correction of motion artefacts while preserving the underlying electrophysiological signals, thereby improving the fidelity of optical mapping measurements obtained from contracting hearts.

### Post‐processing and filtering

After motion correction, optical mapping videos underwent a series of post‐processing steps. These included spatial smoothing using a 5 × 5 box filter, temporal smoothing with a 5‐frame moving average filter, and baseline drift correction using a moving average filter with a window length corresponding to the cardiac cycle length.

MAP recordings, originally acquired at a sampling rate of 1 kHz, were down‐sampled to 250 Hz to match the temporal resolution of the optical recordings and enable direct comparison. The MAP signals were subsequently processed using the same temporal filtering and baseline correction procedures applied to the optical data.

### Electrophysiological analysis and validation of motion‐correction accuracy

To assess the accuracy of the motion‐correction approach, OAP signals were compared with simultaneously recorded MAP signals. Because the MAP electrode occupied a portion of the optical field of view, optical signals could not be extracted from the exact electrode location. Instead, OAP signals obtained from a neighbouring region (approximately 3–4 mm away) were used for comparison.

Action potential durations (APD_50_, APD_70_ and APD_80_) were computed as the time interval between activation time, defined as the time of the maximum rate of voltage change (d*V*/d*t*_max_), and the points of 50%, 70% and 80% repolarization, respectively. APD_90_ was not analysed because residual motion artefacts near the baseline during late repolarization limited reliable measurement in some preparations. Optical APD values were obtained by averaging APD measurements from three pixels adjacent to the MAP electrode.

APD served as the primary quantitative metric for direct comparison between MAP and OAP signals to validate the accuracy of motion correction. For paced and sinus rhythm recordings, activation and APD maps were generated from optical mapping data to visualize the effects of motion correction on spatiotemporal activation and repolarization patterns. For VF recordings, dominant frequency maps were computed using Welch power spectral density estimation (Berenfeld et al., 2000). Power spectral density was estimated using a Hann window, 512‐sample segments, 50% overlap and a 512‐point fast Fourier transform at a sampling frequency of 250 Hz, giving a frequency resolution of 0.488 Hz. Dominant frequency was defined as the frequency with the highest spectral power.

### Statistical analysis

Agreement between optical and MAP APD measurements was evaluated using Bland–Altman analysis, with bias defined as MAP − OAP and limits of agreement calculated as mean bias ± 1.96 SD of the differences. The 95% confidence intervals were estimated for the bias and limits of agreement. Measurement precision was further assessed using empirical cumulative distribution functions, representing cumulative probability curves, of the absolute differences between optical and MAP APD values, from which the proportion of measurements within predefined agreement thresholds (e.g. ±10 ms) was determined. Bland–Altman and cumulative probability analyses included all individual APD measurements without temporal averaging. Due to the limited number of hearts recorded using RH237, data from Di‐4‐ANEPPS‐ and RH237‐stained hearts were pooled, yielding a total sample size of seven hearts (*n* = 7), comprising five Di‐4‐ANEPPS and two RH237 preparations.

## Results

### Effect of motion correction on optical action potential time traces

To assess the impact of motion correction on OAP morphology, signals were compared across four processing conditions: prior to motion tracking or ratiometry, after motion tracking alone, after ratiometry alone, and following the combined application of motion tracking and ratiometry. Individually, motion tracking or ratiometry improved signal quality, whereas their combined application produced the most stable and physiologically interpretable waveforms across regions exhibiting different degrees of motion.

**Figure 2:**
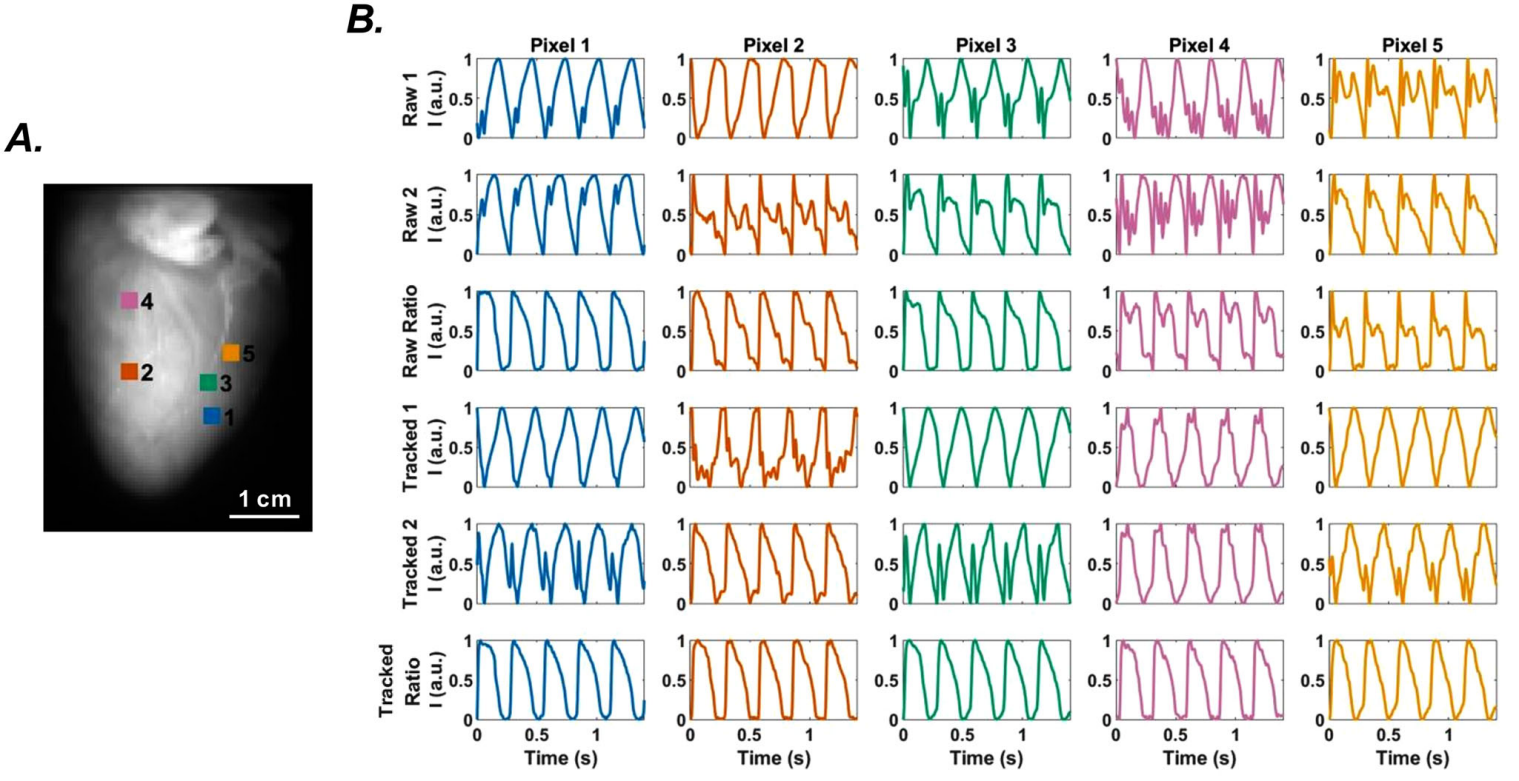
Motion correction using combined motion tracking and ratiometry in a contracting rabbit heart *A*, representative video frame from optical mapping of the left ventricular epicardial surface showing five randomly selected sites (pixels 1–5). *B*, optical signals recorded from each site (pixels 1–5) during pacing at a cycle length of 280 ms. Traces show raw dual‐camera recordings (Raw 1 and Raw 2), the ratio computed from the raw signals (Raw Ratio), signals following motion tracking (Tracked 1 and Tracked 2), and the ratio computed after motion tracking (Tracked Ratio). The Tracked Ratio signal represents the fully motion‐corrected optical action potential.

Representative examples are shown in Fig. 2 from a contracting rabbit heart paced at a cycle length of 280 ms. Fig. 2*A* shows a representative video frame of the left ventricular epicardial surface with five randomly selected sites (pixels 1–5) used for signal extraction, and Fig. 2*B* displays the corresponding optical signals recorded at these locations.

At individual locations, motion tracking or ratiometry applied separately reduced motion‐related distortion to varying extents. However, their effectiveness depended on local mechanical dynamics and did not consistently yield reliable action potential morphology across the heart. In contrast, the combined motion‐correction strategy markedly suppressed residual artefacts, producing OAP traces with smoother morphology and enhanced temporal stability across all sampled locations.

### Action potential propagation and duration before and after motion correction

**Figure 3:**
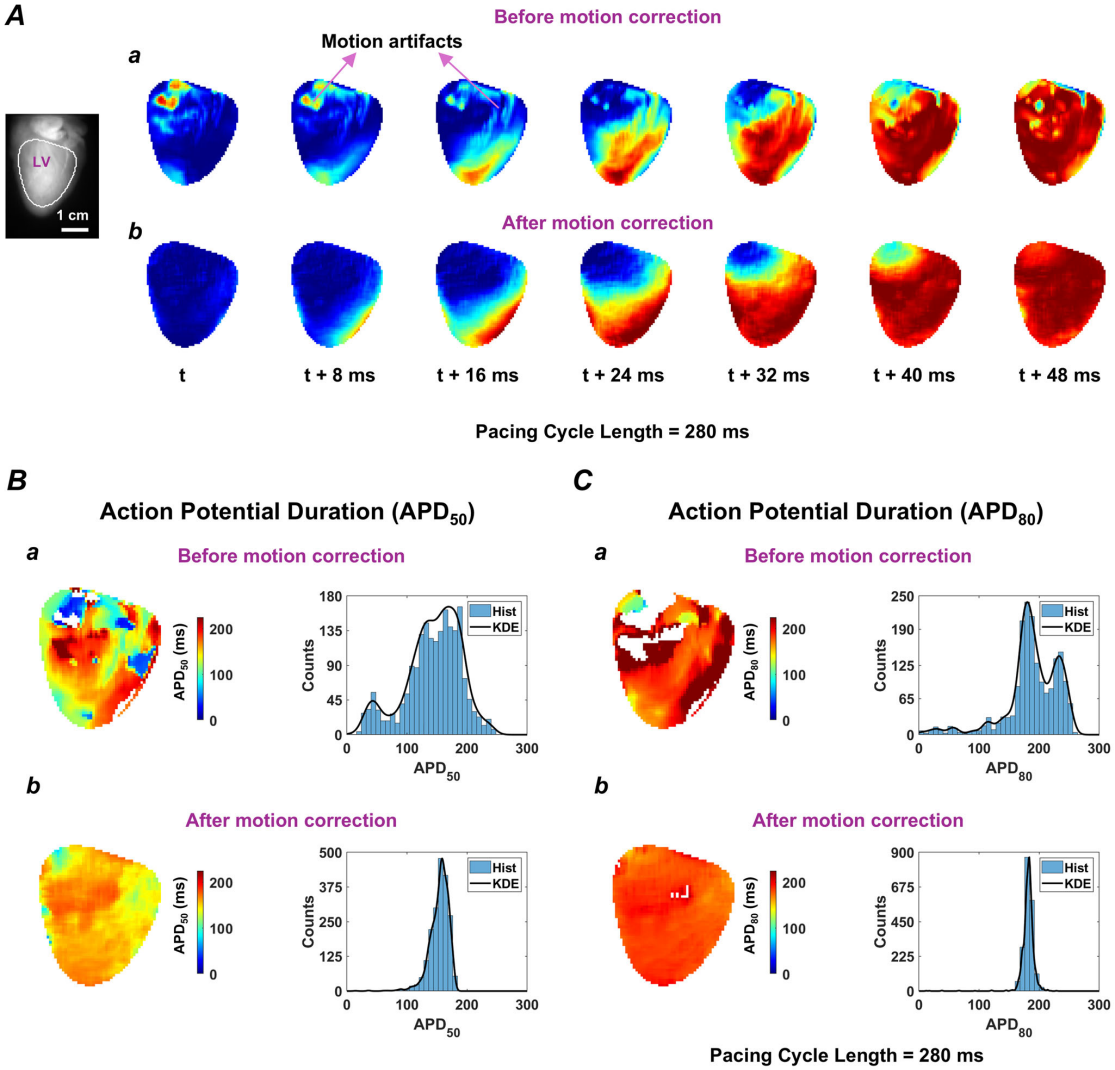
Effect of motion correction on wave propagation and action potential duration (APD) measurements *A*, representative wave propagation snapshots obtained from optical mapping videos during a right ventricular (RV) paced beat, with pacing delivered from the mid‐RV region located on the posterior side of the imaged surface. *a*, wave propagation before motion correction, where motion artefacts appear as high‐frequency network‐like spatial patterns and spurious excitation signals superimposed on and distorting the action potential wave pattern. *b*, wave propagation after motion correction, with marked reduction of motion artefacts and clearer wavefront structure. *B*, action potential duration at 50% repolarization (APD_50_). *a*, APD_50_ map and corresponding distribution visualized using histograms and kernel density estimation before motion correction. *b*, APD_50_ map and distribution after motion correction. Prior to correction, APD_50_ maps exhibit pronounced spatial heterogeneity with a broad distribution, whereas motion correction results in more homogeneous APD_50_ maps and a narrower distribution. *C*, action potential duration at 80% repolarization (APD_80_), presented using the same layout and analysis as in *B*. The region of interest used for wave propagation and APD analyses is shown in the top left of panel *A*.

Motion artefacts substantially influence the visualization of electrical activity and the quantification of electrophysiological parameters during optical mapping of contracting hearts. The effects of motion correction on wave propagation and APD measurements are summarized in Fig. 3 for a rabbit heart paced at a cycle length of 280 ms.

Prior to motion correction, propagation patterns were distorted by motion‐induced artefacts, visible as high‐frequency spatial structures and spurious excitation signals superimposed on the action potential wavefront (Fig. 3*Aa*). These distortions obscured the activation sequence and reduced clarity of conduction patterns. Following motion correction, wavefronts appeared smoother and more continuous, enabling clearer visualization of activation dynamics (Fig. 3*Ab*).

APD_50_ and APD_80_ maps showed marked spatial heterogeneity before motion correction (Fig. 3*Ba*,*Ca*). Histogram and kernel density analyses demonstrated broad APD distributions, consistent with increased variability. After motion correction, APD maps became more spatially uniform, with smoother regional patterns and reduced apparent dispersion (Fig. 3*Bb*,*Cb*). Correspondingly, APD distributions narrowed, indicating reduced variability and improved measurement consistency. The effects of motion artefacts were more pronounced during repolarization, reflected in APD measurements, than during depolarization associated with wave propagation.

### Influence of motion correction on dominant frequency analysis during ventricular fibrillation

**Figure 4:**
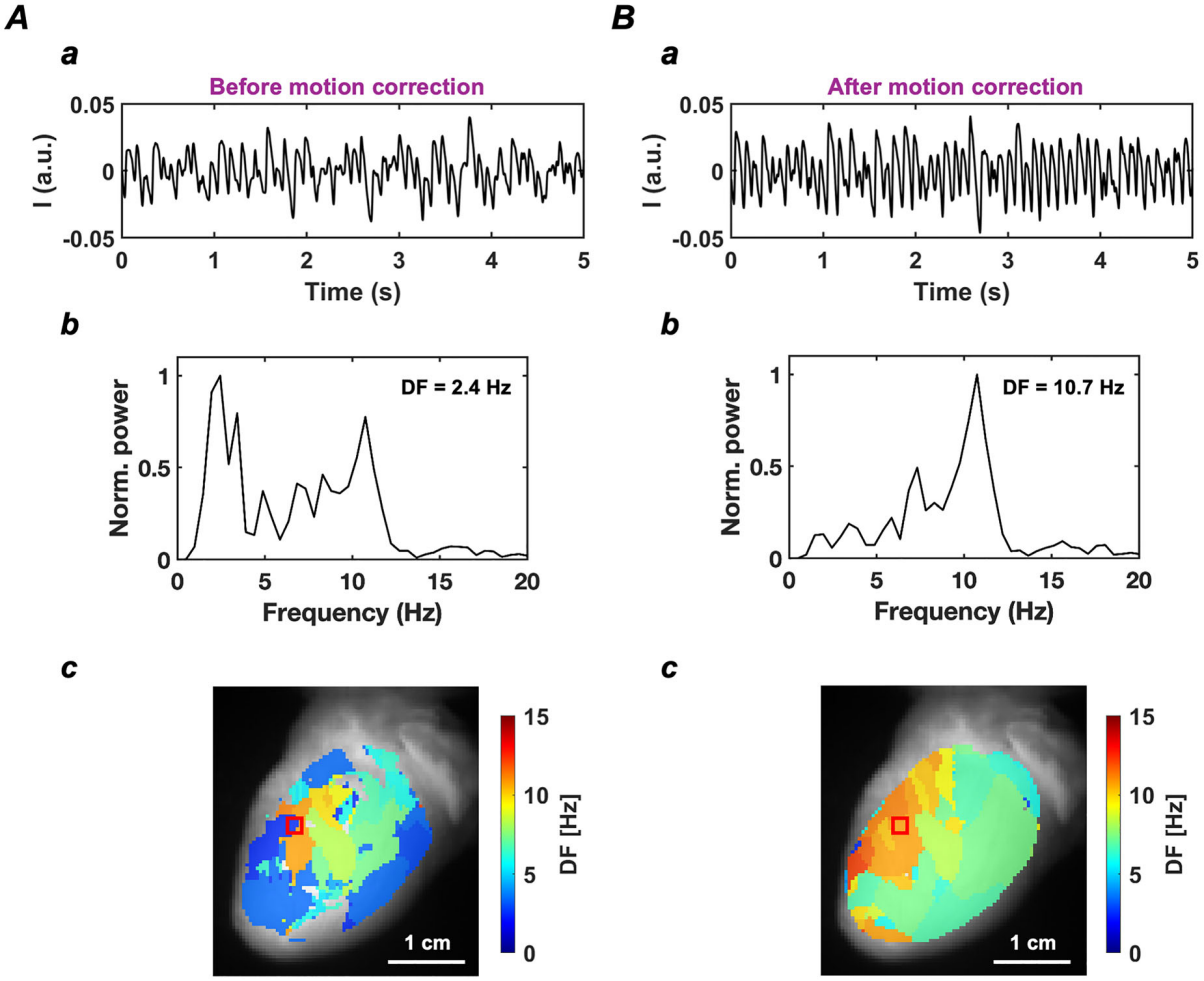
Dominant frequency of ventricular fibrillation before and after motion correction *A*, ventricular fibrillation recordings before motion correction. *a*, representative ventricular fibrillation (VF) time traces. *b*, corresponding normalised Welch‐derived power spectra. *c*, dominant frequency map overlaid on a representative single‐frame heart image. *B*, ventricular fibrillation recordings after motion correction. *a*, VF time traces. *b*, corresponding normalised Welch‐derived power spectra. *c*, dominant frequency map after correction.

Motion artefacts influence the measured frequency characteristics of VF, leading to variability in dominant frequency estimates. The effects of motion correction on VF frequency variation are summarized in Fig. 4. In uncorrected recordings, VF time‐domain traces displayed pronounced signal distortion, characterized by irregular waveform morphology and reduced signal integrity (Fig. 4*Aa*). These distortions obscured the underlying electrophysiological activity and reduced clarity of VF dynamics. Following motion correction, motion‐induced artefacts were reduced, and VF signals exhibited smoother waveforms with more temporally consistent behaviour (Fig. 4*Ba*), enabling clearer visualization of VF activity.

Corresponding changes were observed in the frequency domain. Power spectra obtained before motion correction were dominated by low‐frequency components, consistent with contamination by motion‐related artefacts (Fig. 4*Ab*). After motion correction, spectral content shifted toward higher frequencies with more clearly defined dominant frequency peaks (Fig. 4*Bb*). In the representative example, the dominant frequency increased from 2.4 Hz before motion correction to 10.7 Hz after motion correction.

Dominant frequency maps further reflected these differences. Prior to motion correction, maps showed predominantly low‐frequency values and spatial irregularities attributable to cardiac motion (Fig. 4*Ac*). After motion correction, higher dominant frequency values and more spatially coherent patterns were observed (Fig. 4*Bc*). Together, these findings indicate improved time‐domain and frequency‐domain characterization of VF following motion correction. These findings indicate that motion correction reduces motion‐induced bias in VF frequency measurements and improves the stability of dominant frequency estimation.

### Comparison of optical and monophasic action potentials before and after motion correction

**Figure 5:**
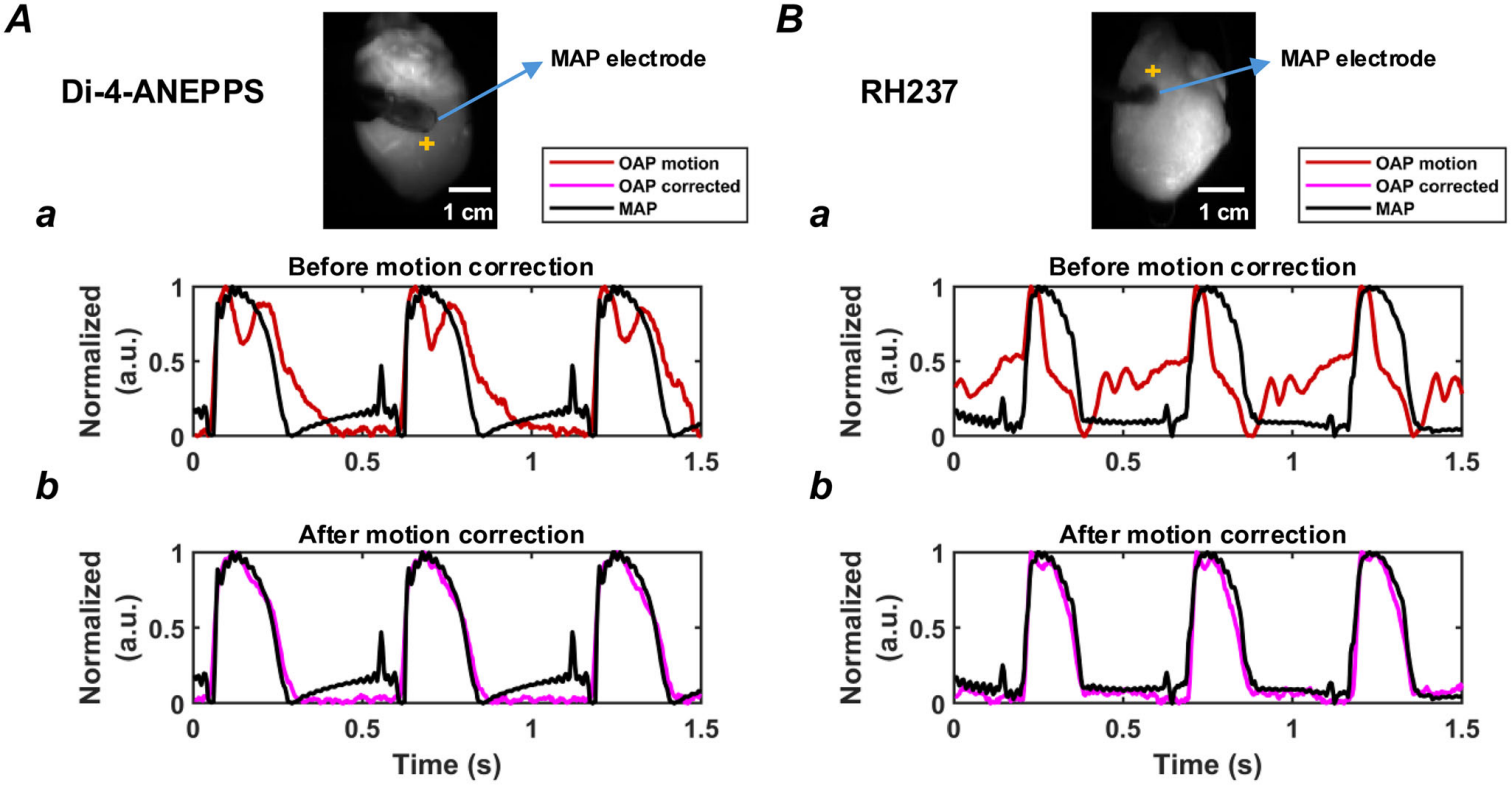
Representative optical action potentials (OAPs) and monophasic action potentials (MAPs) recorded from a rabbit heart during sinus rhythm using Di‐4‐ANEPPS and RH237 *A*, overlay of MAP and OAP traces for Di‐4‐ANEPPS before (*a*), and after (*b*), motion correction. OAP signals are shown in red before correction and magenta after correction, while MAP traces are shown in black. Prior to correction, OAPs exhibit substantial distortion and deviate markedly from the MAPs. After motion correction, OAP signals show reduced distortion and improved agreement with MAPs in waveform morphology and timing. *B*, overlay of OAP and MAP traces for RH237 before (*a*), and after (*b*), motion correction, using the same colour scheme as in *A*. Motion‐corrected OAPs show clearer morphology and closer temporal correspondence with MAP signals. Corresponding optical single frames indicating the MAP electrode location are shown at the top of panels *A* and *B*. The optical recording site is indicated by an orange plus symbol (+). Optical traces were extracted from pixels approximately 4 mm from the electrode, as signals recorded directly at the electrode position are distorted.

To evaluate the effect of motion correction on optical signal fidelity, OAPs were compared with simultaneously recorded MAPs during sinus rhythm (Fig. 5). For recordings obtained using Di‐4‐ANEPPS, pronounced distortion of the OAP waveform was observed before motion correction, with substantial deviations from the corresponding MAP in both morphology and timing (Fig. 5*Aa*). The optical upstroke remained relatively preserved, whereas larger discrepancies were evident during the repolarization phase.

After motion correction, Di‐4‐ANEPPS OAPs showed marked improvement in signal quality (Fig. 5*Ab*). Corrected optical waveforms closely matched MAPs, demonstrating improved agreement in morphology and temporal alignment, consistent with reduced motion‐related artefacts. Similar findings were observed with RH237 recordings. Before motion correction, RH237 OAPs exhibited distorted waveforms and poor correspondence with MAP signals (Fig. 5*Ba*), whereas motion‐corrected signals showed clearer morphology and closer temporal agreement with MAPs (Fig. 5*Bb*).

Together, these findings show improved agreement between optical and electrical action potential recordings following motion correction for both voltage‐sensitive dyes.

### Validation of motion‐corrected optical APD measurements: agreement and precision relative to map recordings

To evaluate the accuracy of motion‐corrected optical mapping for quantifying action potential duration, optical APD measurements were compared with simultaneously recorded MAPs across different processing conditions during sinus rhythm. Agreement between modalities was assessed using Bland–Altman analysis, per‐heart APD_80_ comparisons and per‐heart MAP–OAP differences, while cumulative probability analysis was used to examine the precision of optical APD measurements relative to MAP recordings.

### Agreement between map and optical APD measurements

**Figure 6:**
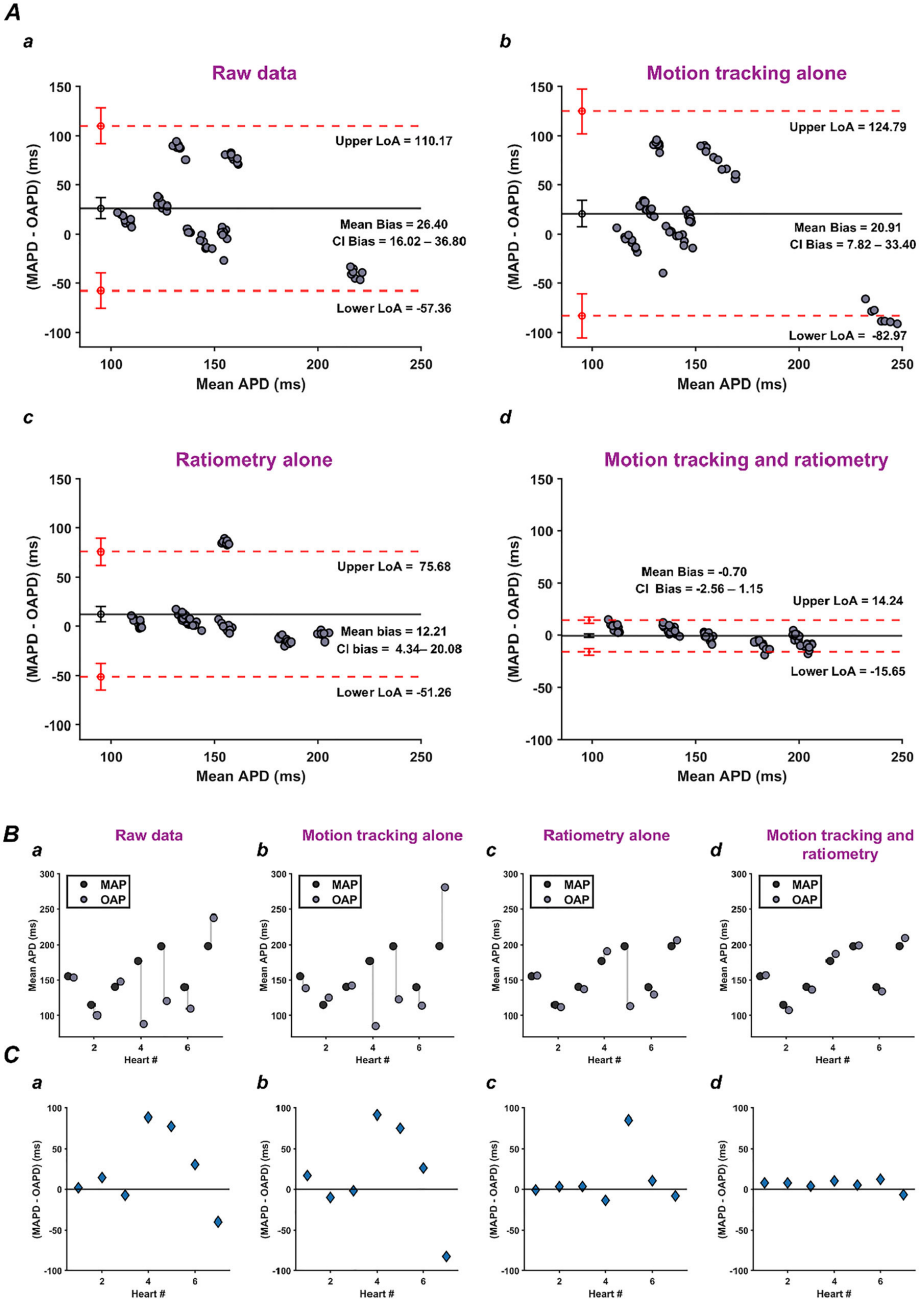
Comparison of monophasic action potentials (MAPs) and optical action potentials (OAPs) across different motion‐correction conditions *A*, Bland–Altman analysis of APD_80_ measurements obtained from seven hearts (*N* = 7), with differences plotted as MAP−OAP. The solid black line indicates the mean bias, while red dashed lines denote the upper and lower limits of agreement (LoA). Black error bars represent the 95% confidence interval (CI) of the bias, and red error bars indicate the 95% CI of the LoA. Subpanels correspond to *a*, raw data, *b*, motion tracking alone, *c*, ratiometry alone and *d*, combined motion tracking and ratiometry. MAPD and OAPD denote action potential duration (APD) measured from MAP and optical recordings, respectively. MAPD and OAPD values cluster closely in the combined motion tracking and ratiometry condition, with differences centred near zero and the CI of the bias including zero, whereas the other conditions exhibit a broader spread and systematic differences between MAP and OAP measurements. *B*, mean APD_80_ values per heart derived from MAP and OAP recordings for each processing condition (*a*–*d*, as in panel *A*). Mean MAPD and OAPD values show close overlap in the combined motion tracking and ratiometry condition, while greater separation between modalities is observed in the raw, motion tracking‐alone and ratiometry‐alone conditions. *C*, per‐heart APD_80_ differences (MAPD−OAPD) for each processing condition. Differences are more tightly distributed in the combined motion tracking and ratiometry condition, whereas raw data, motion tracking alone and ratiometry alone exhibit a broader spread between MAP and OAP measurements.

Agreement between MAP and OAP APD_80_ measurements across motion‐correction conditions is summarized in Fig. 6, which includes Bland–Altman analysis and per‐heart comparisons of APD values and differences between MAP and OAP measurements.

Bland–Altman analysis (Fig. 6*A*) demonstrated improved agreement following combined motion tracking and ratiometry compared with raw data or either method applied alone. In the fully corrected condition, the mean bias was ‐0.70 ms, with limits of agreement ranging from ‐15.65 ms to 14.24 ms, and the confidence interval of the bias included zero, indicating the absence of systematic bias between MAP and OAP APD_80_ measurements. In contrast, the confidence intervals of the bias for the raw data, motion tracking alone, and ratiometry‐alone conditions did not include zero, indicating systematic bias between MAP and OAP APD_80_ measurements under those processing conditions. Raw data exhibited a mean bias of 26.40 ms with limits of agreement from ‐57.36 to 110.17 ms, motion tracking alone showed a mean bias of 20.91 ms with limits of agreement from ‐82.97 to 124.79 ms, and ratiometry alone demonstrated a mean bias of 12.21 ms with limits of agreement from ‐51.26 to 75.68 ms, reflecting larger systematic differences and broader variability compared with the combined motion‐correction approach.

**Figure A2:**
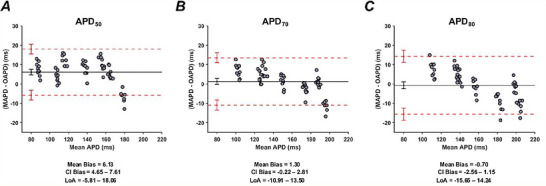
Bland–Altman analysis of agreement between monophasic action potentials (MAPs) and motion‐corrected optical action potentials (OAPs) for APD_50_, APD_70_ and APD_80_ under the combined motion tracking and ratiometry condition Bland–Altman analyses were performed using measurements obtained from seven hearts (*N* = 7). Differences are plotted as MAP − OAP. The mean bias is shown as a solid black line, with its 95% confidence interval (CI) indicated by black error bars. The upper and lower limits of agreement (LoA) are shown as dashed red lines, with their corresponding 95% confidence intervals indicated by red error bars. *A*–*C*, Bland–Altman plots for APD_50_, APD_70_ and APD_80_, respectively. *C*, expanded view of the APD_80_ Bland–Altman plot; the same analysis is shown with a common axis scale across correction methods in Fig. 6*Ad*.

Bland–Altman analyses were also performed for APD_50_ and APD_70_ to assess agreement during earlier phases of repolarization. Corresponding plots for the combined motion tracking and ratiometry condition are shown in Fig. A2*A,B*, respectively. APD_50_ showed a small systematic bias, as the 95% confidence interval of the mean bias did not include zero, whereas no systematic bias was observed at APD_70_, where the confidence interval of the bias included zero.

Fig. A2*C* shows a zoomed Bland–Altman plot for APD_80_ under the same condition to facilitate visualization of the distribution of differences, as the APD_80_ plot in Fig. 6*Ad* uses a wider axis range to allow direct comparison across motion‐correction conditions.

Comparison of per‐heart APD_80_ measurements (Fig. 6*B*) showed close overlap between MAP and OAP values in the combined motion tracking and ratiometry condition, whereas greater separation between modalities was evident in raw data and in motion tracking alone or ratiometry‐alone conditions. Per‐heart APD_80_ differences (Fig. 6*C*) were more tightly distributed around zero following combined motion correction, while substantially broader dispersion was observed in the other processing conditions, reflecting increased variability between MAP and optical measurements in the absence of integrated motion correction.

### Cumulative probability analysis of differences between map and optical APD measurements

**Figure 7:**
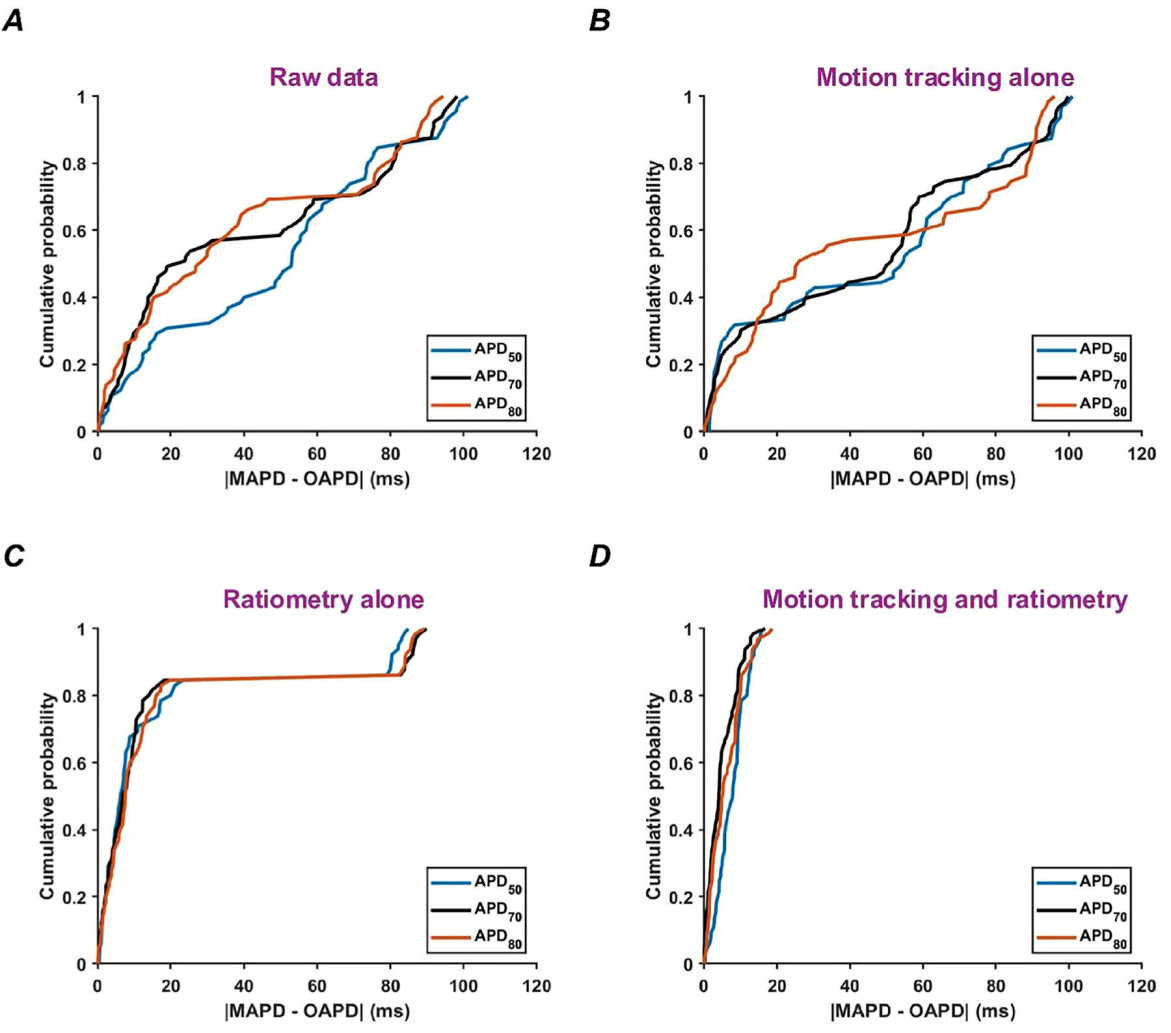
Cumulative probability analysis of absolute differences between monophasic action potential (MAP) and optical action potential (OAP) action potential duration (APD) measurements across motion‐correction conditions Cumulative probability curves show the proportion of measurements within increasing absolute agreement thresholds (|MAP − OAP|) for APD_50_, APD_70_ and APD_80_. Panels correspond to *A*, raw data, *B*, motion tracking alone, *C*, ratiometry alone and D, combined motion tracking and ratiometry. Curves in the combined motion tracking and ratiometry condition are shifted toward the upper left, indicating a greater proportion of measurements with smaller absolute differences and improved agreement with MAP APD measurements.

To further assess measurement precision, cumulative probability analysis of absolute differences between MAP and OAP APD measurements was performed across all processing conditions (Fig. 7). Cumulative probability curves demonstrated a marked improvement in precision following combined motion tracking and ratiometry, with the curves in this condition being steeper and shifted toward the upper left, indicating a greater proportion of measurements with small differences between MAP and OAP APD measurements. In contrast, curves obtained from raw data and from motion tracking or ratiometry alone were flatter and shifted toward the right, reflecting larger differences and greater variability between MAP and optical APD measurements. The proportion of optical APD measurements within ±10 ms of MAP increased from 16.9%, 29.2% and 26.2% in raw recordings to 31.7%, 28.6% and 22.2% with motion tracking alone; 67.7%, 66.2%, and 61.5% with ratiometry alone; and 75.4%, 87.7% and 80% after combined motion correction for APD_50_, APD_70_ and APD_80_, respectively. These findings indicate that while motion tracking or ratiometry individually improve agreement to some extent, their combined application yields the greatest enhancement in measurement precision relative to MAP recordings.

## Discussion

Optical mapping of cardiac electrophysiology in contracting hearts is fundamentally challenged by motion‐induced artefacts that distort both temporal waveforms and spatial patterns of electrical activity (Christoph & Luther, 2018; Rohde et al., 2005). In this study, we show that combining marker‐free motion tracking with emission ratiometry enables reliable optical mapping in freely contracting rabbit hearts without the need for mechanical stabilization or pharmacological EC uncoupling. Validation against simultaneously recorded MAPs indicates that the corrected optical signals preserve the underlying electrophysiological information under physiologically relevant conditions.

A central observation of this work is that motion tracking and emission ratiometry address complementary sources of motion artefacts. Motion tracking primarily corrects spatial misregistration arising from tissue displacement, whereas emission ratiometry suppresses shared photometric fluctuations affecting both emission channels. When applied individually, each method mitigates only part of the motion‐related distortion. In the present dataset, this complementarity was also evident quantitatively from the Bland–Altman analysis of APD_80_. Relative to raw contracting optical signals, both correction methods improved agreement with MAP recordings, but ratiometry alone produced a larger reduction in systematic bias than motion tracking alone, reducing the mean bias from 26.40 ms to 12.21 ms compared with 20.91 ms after motion tracking alone. This suggests that, for APD_80_ in this preparation, ratiometry made the larger individual contribution to reducing systematic APD error. However, neither method alone eliminated the residual bias, whereas the combined approach reduced the mean bias further to ‐0.70 ms. These findings support the interpretation that the two approaches are complementary rather than interchangeable. Their combined application therefore provides a more comprehensive correction strategy capable of recovering physiologically interpretable optical signals across the contracting myocardium.

The close correspondence between motion‐corrected OAPs and MAP recordings indicates that the combined correction strategy preserves key electrophysiological features of the cardiac action potential. In particular, the agreement observed for repolarization measurements suggests that optical APD analysis remains reliable even in the presence of active myocardial contraction. The modest systematic bias observed at APD_50_ (mean bias ≈ 6 ms) may reflect the greater mechanical deformation occurring during early repolarization, a phase during which optical measurements may remain more sensitive to mechanical activity.

Beyond improving the fidelity of individual optical signals, effective suppression of motion artefacts has important implications for spatial electrophysiological analyses. Motion‐induced spatial distortions caused by tissue displacement can obscure activation patterns and introduce artificial heterogeneity, complicating interpretation of conduction dynamics and repolarization gradients (Kappadan et al., 2023). By reducing both spatial misregistration and photometric motion artefacts, the combined correction strategy improves the interpretability of optical mapping data while preserving the intrinsic heterogeneity of cardiac electrical activity.

These improvements are particularly relevant for studies of complex arrhythmias such as VF. Motion artefacts can distort optical mapping signals and obscure the underlying activation dynamics of fibrillatory activity (Christoph et al., 2017; Efimov et al., 2004; Kappadan et al., 2020). The present findings suggest that mechanical motion can introduce bias in electrophysiological measurements, including dominant frequency estimation, by contaminating optical signals with low‐frequency artefacts. By reducing motion‐induced distortion, motion correction improves signal interpretability and enables more reliable characterization of the spatiotemporal dynamics underlying VF.

Previous studies have explored various strategies to mitigate motion artefacts during optical mapping of contracting hearts, including marker‐based tracking approaches and ratiometric imaging techniques (Bachtel et al., 2011; Knisley et al., 2000; Zhang et al., 2016, 2023). While these methods have demonstrated partial suppression of motion‐related distortions, systematic validation of marker‐free motion tracking combined specifically with emission ratiometry against an independent electrical reference has been limited. The present study addresses this gap by validating motion‐corrected optical signals against simultaneously recorded MAPs, demonstrating that a fully optical correction strategy can reliably recover electrophysiological signals in actively contracting myocardium.

Beyond the present validation, the ability to perform optical mapping in actively contracting myocardium may broaden the range of experimental questions that can be addressed using optical techniques. Preserving physiological contraction enables investigation of mechano‐electric feedback (Franz, 2000; Quinn & Kohl, 2021), stretch‐induced electrophysiological responses (Taggart & Lab, 2008; Zabel et al., 1996), and the interaction between mechanical deformation and arrhythmogenesis (Franz & Bode, 2003; Nazir & Lab, 1996). These processes are difficult to study using conventional optical mapping approaches that rely on EC uncoupling (Fedorov et al., 2007; Swift et al., 2021). The correction strategy described here therefore provides a framework for investigating cardiac electrophysiology under conditions that more closely resemble the intact beating heart.

In summary, marker‐free motion tracking combined with emission ratiometry provides a practical strategy for optical mapping of electrical activity in contracting hearts. By enabling reliable analysis of action potential morphology, repolarization dynamics, wave propagation and arrhythmia‐related activity without suppressing cardiac motion, this approach advances optical mapping toward more physiologically relevant experimental conditions that preserve intact electromechanical coupling.

### Limitations and future directions

Notwithstanding the methodological advances described above, this study has several limitations. First, the motion‐correction framework is based on 2D optical flow‐based motion tracking, which estimates motion within the image plane of the camera. Although contrast enhancement was applied to reduce the influence of voltage‐dependent fluorescence changes and to improve the robustness of motion estimation, optical flow fundamentally derives a displacement field from changes in image intensity patterns. Consequently, the resulting displacement field represents an image‐derived estimate of tissue motion, rather than a direct measurement of true myocardial tissue displacement. This approach effectively captures in‐plane motion but does not explicitly account for out‐of‐plane motion or three‐dimensional (3D) tissue deformation and is therefore unable to capture the full 3D structural and mechanical dynamics of the heart. This limitation is particularly relevant near the edges of the field of view, where the curved epicardial surface is viewed obliquely and where 2D projection may distort apparent activation patterns, conduction direction or conduction velocity. Accordingly, conduction and repolarization patterns in peripheral regions should be interpreted cautiously, and quantitative analyses in the present study were focused on regions where corrected signals remained physiologically plausible and free from obvious residual distortion. Future integration with panoramic or multi‐view optical mapping systems may help address this limitation by enabling more comprehensive characterization of 3D cardiac structural and mechanical dynamics. Other 3D motion‐tracking approaches, including structured‐light surface imaging or ultrasound‐based motion assessment, may also provide complementary strategies for capturing out‐of‐plane deformation in future studies.

Second, although OAPs and MAPs were recorded simultaneously, they were not obtained from the exact same spatial location. Optical signals were extracted from pixels in close proximity to the MAP electrode to minimize spatial separation; however, small differences in sampling location may contribute to minor discrepancies between optical and MAP‐derived measurements. In addition, some mismatch may reflect limitations inherent in MAP recording itself, which requires stable, firm contact between the electrode and the epicardial surface. Although this contact is necessary to obtain a MAP signal, excessive local pressure could theoretically alter local mechanical conditions, restrict superficial microvascular perfusion and thereby produce regional ischaemic effects beneath the electrode. Moreover, OAPs and MAPs do not sample identical tissue volumes: optical recordings predominantly reflect a relatively shallow epicardial layer, whereas MAP recordings reflect electrical activity arising from a larger local myocardial volume under and around the contact site. These factors may have contributed to the small differences observed between MAPs and nearby OAPs. Despite these limitations, the strong agreement observed across multiple repolarization levels indicates that the comparison provides a robust validation of motion‐corrected optical mapping.

Third, ratiometric optical mapping increases system complexity. In the case of emission ratiometry, simultaneous acquisition of two emission signals typically requires two synchronized cameras and additional optical components, increasing hardware cost and experimental complexity. Importantly, this limitation is not unique to emission ratiometry. Excitation ratiometry also introduces hardware and software complexity, as it requires rapid excitation wavelength switching and precise synchronization between light source control and image acquisition. Future developments in optical instrumentation, such as advanced multiplexed detection schemes that enable simultaneous acquisition of multiple optical channels with reduced detector hardware, as well as computational approaches that infer ratio‐like signals from single‐camera recordings, may help mitigate these constraints and improve the practicality of ratiometric optical mapping.

Fourth, experiments were performed in unloaded, non‐PV‐working Langendorff‐perfused hearts. This preparation does not reproduce the PV work of the intact heart and is likely to operate at a lower metabolic demand than PV‐working preparations (Wengrowski et al., 2014). As metabolic state and mechanical loading both influence cardiac electrophysiology, including mechano‐electric feedback, extension of the present framework to PV‐working preparations presents an important direction for future work.

Finally, a limitation of the present study is the absence of a simple, objective, one‐step method for defining which regions are sufficiently reliable for quantitative electrophysiological analysis after motion correction. Although the combination of motion tracking and ratiometry substantially reduced motion artefact, the final selection of analysable regions still required several pragmatic quality‐control steps, including conservative exclusion of peripheral regions, visual review of corrected signals across multiple myocardial regions, and exclusion of areas where APD calculation was unreliable or physiologically implausible. This reflects a broader technical challenge in contracting‐heart optical mapping, where even small residual motion can distort OAPs and calcium transients, and where motion correction remains difficult in the presence of boundary effects, out‐of‐plane deformation and spatially heterogeneous tissue motion. In the present study, this pragmatic approach was considered appropriate because the preparations were predominantly healthy hearts, in which large pathological APD and mechanical heterogeneity were not expected. However, the approach remains partly operator‐dependent and may introduce greater uncertainty in diseased preparations, where true regional electrophysiological and mechanical heterogeneity may be more pronounced and therefore harder to distinguish from residual tracking uncertainty or motion‐related artefact. Future studies would benefit from objective pixel‐wise motion‐quality metrics, confidence maps or automated criteria that integrate tracking reliability, residual motion artefact and signal or APD quality into a reproducible assessment of whether each region is suitable for quantitative analysis.

## Conclusion

This study presents a validated framework for optical mapping of cardiac electrical activity in contracting hearts based on the combined use of marker‐free motion tracking and emission ratiometry, with systematic validation using MAPs. By addressing both motion‐induced spatial misregistration and common‐mode optical artefacts, this approach enables stable and interpretable optical recordings under conditions that preserve normal cardiac contraction.

Comparison with simultaneously recorded MAPs shows that motion‐corrected optical signals preserve overall action potential waveform morphology while accurately capturing repolarization timing. The framework further supports reliable analysis of electrical wave propagation, action potential duration and arrhythmic activity in the presence of active motion.

By enabling optical mapping without mechanical stabilization or EC uncoupling, this work advances the methodology toward more physiologically relevant experimental conditions and broadens the applicability of optical mapping for studying cardiac electrophysiology in intact contracting hearts.

## Additional information

## Competing interests

Jean‐Baptiste Guichard reports honoraria as a lecturer from Microport CRM and Abbott, and unrestricted fellowship grant support from Abbott. He is also a shareholder of CorifyCare SL. All other authors declare no competing interests.

## Author contributions

V.K., Z.H. and F.S.N. conceived and designed research. V.K., J.B.T., N.M., Z.H., P.E., Y.A., X.B.S. and J.‐B.G. performed the experiments. J.L. and J.C. provided the library for motion tracking. V.K. analysed the data, prepared the figures and drafted the manuscript. D.A.‐J. and A.S. contributed to the experimental design and development of the hardware platform used in this study. V.K., Z.H., J.B.T., J.L., J.C., N.S.P. and F.S.N. edited and revised the manuscript. All authors have read and approved the final version of the manuscript.

## Funding

This study was supported by the British Heart Foundation RE/24/130023 and RG/F/22/110078 (to F.S.N., N.S.P. and V.K.), Imperial Post‐Doctoral, Post‐CCT Research Fellowship IPPRF MED04970 (to J.T.) and Contractes d'Investigació Avançada Fundació BBVA – Hospital Clínic Barcelona Joan Rodés – Josep Baselga 2022 (HCB_BIO_001/2) and Research Grant Daniel Bravo 2024 – Foundation Daniel Bravo (J.‐B.G).

## Supporting information


Peer Review History

## Data Availability

The data supporting the findings of this study are available from the corresponding author upon reasonable request. Code and tutorials for the optimap library are available at the following link: https://cardiacvision.github.io/optimap/main/tutorials/
