# Supplementary material for: Combined emission ratiometry and motion tracking for optical mapping of contracting hearts: Validation with monophasic action potentials
Source: J Physiol. 2026 Jun 16;604(14):5758–76. doi: 10.1113/JP291400 (PMC13370694; doi:10.1113/JP291400)
Supplement: Supplementary file 1 — Peer Review History [file TJP-604-5758-s001.pdf]

The Journal of Physiology

Combined Emission Ratiometry and Motion Tracking for Optical Mapping of Contracting Hearts: Validation with Monophasic Action Potentials

Vineesh Kappadan, Zhen Hua, Johanna B Tonko, Najmah Mohamed, Jan Lebert, Paraskevas Efstathiou, Yasser Abdelghani, Danya Agha-Jaffar, Anies Sohi, Xianbo Sun, Jean-Baptiste Guichard, Jan Christoph, Nicholas S Peters, and Fu Siong Ng
DOI: 10.1113/JP291400

Corresponding author(s): Fu Siong Ng (f.ng@imperial.ac.uk)

The following individual(s) involved in review of this submission have agreed to reveal their identity: Matthew W Kay (Referee #1); Igor R. Efimov (Referee #2)

Review Timeline:

Submission Date:	20-Mar-2026
Editorial Decision:	08-Apr-2026
Revision Received:	05-May-2026
Accepted:	15-May-2026

Senior Editor: Eleonora Grandi

Reviewing Editor: T Alexander Quinn

Transaction Report:

(Note: With the exception of the correction of typographical or spelling errors that could be a source of ambiguity, letters and reports are not edited. Depending on transfer agreements, referee reports obtained elsewhere may or may not be included in this compilation. Referee reports are anonymous unless the Referee chooses to sign their reports.)

Dear Dr Ng,

Re: JP-TFP-2026-291400 "Combined Emission Ratiometry and Motion Tracking for Optical Mapping of Contracting Hearts: Validation with Monophasic Action Potentials" by Vineesh Kappadan, Zhen Hua, Johanna B Tonko, Najmah Mohamed, Jan Lebert, Paraskevas Efstathiou, Yasser Abdelghani, Danya Agha-Jaffar, Anies Sohi, Xianbo Sun, Jean-Baptiste Guichard, Jan Christoph, Nicholas S Peters, and Fu Siong Ng

Thank you for submitting your manuscript to The Journal of Physiology. It has been assessed by a Reviewing Editor and by 2 expert referees and we are pleased to tell you that it is potentially acceptable for publication following satisfactory major revision.

Please advise your co-authors of this decision as soon as possible.

The referee reports are copied at the end of this email.

Please address all the points raised and incorporate all requested revisions or explain in your Response to Referees why a change has not been made. We hope you will find the comments helpful and that you will be able to return your revised manuscript within 9 months. If you require longer than this, please contact journal staff: jp@physoc.org. Please note that this letter does not constitute a guarantee for acceptance of your revised manuscript.

Your revised manuscript should be submitted online using the link in your Author Tasks: Link Not Available. This link is accessible via your account as Corresponding Author; it is not available to your co-authors. If this presents a problem, please contact journal staff (jp@physoc.org). Image files from the previous version are retained on the system. Please ensure you replace or remove any files that are being revised.

If you do not wish to submit a revised version of your manuscript, you must inform our journal staff (jp@physoc.org) or reply to this email to request withdrawal. Please note that a manuscript must be formally withdrawn from the peer review process at one journal before it may be submitted to another journal.

TRANSPARENT PEER REVIEW POLICY: To improve the transparency of its peer review process, The Journal of Physiology publishes online as supporting information the peer review history of all articles accepted for publication. Readers will have access to decision letters, including Editors' comments and referee reports, for each version of the manuscript, as well as any author responses to peer review comments. Referees can decide whether or not they wish to be named on the peer review history document.

ABSTRACT FIGURES: Authors are expected to use The Journal's premium BioRender account to create/redraw their Abstract Figures. Information on how to access this account is here: https://physoc.onlinelibrary.wiley.com/journal/14697793/biorender-access.

This will enable Authors to create and download high-resolution figures. If authors have used the free BioRender service, they can use the instructions provided in the link above to download a high-resolution version suitable for publication.

The link provided should only be used for the purposes of this submission. Authors will be charged for figures created on this account if they are not related to this manuscript submission.

LANGUAGE EDITING AND SUPPORT FOR PUBLICATION: If you would like help with English language editing, or other article preparation support, Wiley Editing Services offers expert help, including English Language Editing, as well as translation, manuscript formatting, and figure formatting at www.wileyauthors.com/eeo/preparation. You can also find resources for Preparing Your Article for general guidance about writing and preparing your manuscript at www.wileyauthors.com/eeo/prepresources.

RIGOUR AND REPRODUCIBILITY: We recommend authors consult The Journal's Rigour and Reproducibility requirements to ensure that their manuscript is suitable for publication: https://physoc.onlinelibrary.wiley.com/pb-assets/hub-assets/physoc/documents/TJP-Rigour-and-Reproducibility-Requirements.pdf

REVISION CHECKLIST:

Check that your Methods section conforms to journal policy: https://jp.msubmit.net/cgi-bin/main.plex?form_type=display_requirements#methods.

Check that data presented conforms to the statistics policy: https://jp.msubmit.net/cgi-bin/main.plex?form_type=display_requirements#statistics.

Upload a full Response to Referees file. To create your 'Response to Referees': copy all the reports, including any comments from the Senior and Reviewing Editors, into a Microsoft Word, or similar, file and respond to each point, using font or background colour to distinguish comments and responses and upload as the required file type.

Please upload two versions of your manuscript text: one with all relevant changes highlighted and one clean version with no changes tracked. The manuscript file should include all tables and figure legends, but each figure/graph should be uploaded as separate, high-resolution files. The journal is now integrated with Wiley's Image Checking service. For further details, see: https://www.wiley.com/en-us/network/publishing/research-publishing/trending-stories/upholding-image-integrity-wileys-image-screening-service

You may also upload:

- 'Potential Cover Art' for consideration as the issue's cover image
- Appropriate Supporting Information (Video, audio or data set: see https://jp.msubmit.net/cgi-bin/main.plex?form_type=display_requirements#supp).

We look forward to receiving your revised submission.

If you have any queries, please reply to this email and we will be pleased to advise.

Yours sincerely,

Eleonora Grandi
Senior Editor
The Journal of Physiology

REQUIRED ITEMS

- Include a Key Points list in the article itself, before the Abstract.

- Author photo and profile. First or joint first authors are asked to provide a short biography (no more than 100 words for one author or 150 words in total for joint first authors) and a portrait photograph. These should be uploaded and clearly labelled together in a Word document with the revised version of the manuscript. See Information for Authors for further details.

- You must start the Methods section with a paragraph headed Ethical approval (https://jp.msubmit.net/cgi-bin/main.plex?form_type=display_requirements#methods).

Research must comply with The Journal's policies regarding animal experiments (https://physoc.onlinelibrary.wiley.com/hub/animal-experiments) and adherence to these policies must be stated in the manuscript.

Authors should confirm in their Methods section that their experiments were carried out according to the guidelines laid down by their institution's animal welfare committee, including an ethics approval reference number. The Methods section must contain a statement about access to food, water and housing, details of the anaesthetic regime: anaesthetic used, dose and route of administration, and method of killing the experimental animals.

- Papers must comply with the Statistics Policy: https://jp.msubmit.net/cgi-bin/main.plex?form_type=display_requirements#statistics.

In summary:

- If n {less than or equal to} 30, all data points must be plotted in the figure in a way that reveals their range and distribution. A bar graph with data points overlaid, a box and whisker plot or a violin plot (preferably with data points included) are acceptable formats.

- If n > 30, then the entire raw dataset must be made available either as supporting information, or hosted on a not-for-profit repository, e.g. FigShare, with access details provided in the manuscript.

- 'n' clearly defined (e.g. x cells from y slices in z animals) in the Methods. Authors should be mindful of pseudoreplication.

- All relevant 'n' values must be clearly stated in the main text, figures and tables.

- The most appropriate summary statistic (e.g. mean or median and standard deviation) must be used. Standard Error of the Mean (SEM) alone is not permitted.

- Exact p values must be stated. Authors must not use 'greater than' or 'less than'. Exact p values must be stated to three significant figures even when 'no statistical significance' is claimed.

- Please include an Abstract Figure file and an Abstract Figure legend. An appropriate figure legend, which should not exceed 150 words in length, should be included in the main manuscript file. The Abstract Figure is a piece of artwork designed to give readers an immediate understanding of the research and should summarise the main conclusions. If possible, the image should be easily 'readable' from left to right or top to bottom. It should show the physiological relevance of the manuscript so readers can assess the importance and content of its findings. Abstract Figures should not merely recapitulate other figures in the manuscript. Please try to keep the diagram as simple as possible and without superfluous information that may distract from the main conclusion(s). Abstract Figures must be provided by authors no later than the revised manuscript stage and should be uploaded as a separate file during online submission labelled as File Type 'Abstract Figure'. Please also ensure that you include the figure legend in the main article file. All Abstract Figures should be created using BioRender. Authors should use The Journal's premium BioRender account to export high-resolution images. Details on how to use and access the premium account are included as part of this email.

- Please ensure that all figures and tables have a title and legend, and that they have been cited within the main article text.

EDITOR COMMENTS

Reviewing Editor:

You paper has been reviewed by two experts in the field, who felt it was a well-written methods paper that would be a useful addition to the literature. They each raised concerns that need to be addressed, which are outlined in the Comments for the Author below. If you feel you can address their concerns, please submit a revised version of your paper, along with a point-by-point response to the reviewers.

Senior Editor:

I concur with the Reviewing Editor's assessment and recommendation.

REFEREE COMMENTS

Referee #1:

This is an important methods manuscript that provides a robust and relatively comprehensive analysis of non-contact optical mapping of action potentials from the epicardium of contracting unloaded perfused rabbit hearts. Ratiometric motion-tracked optical action potentials are compared to those of a contact measurement, the monophasic action potential, to demonstrate the great utility of this optical mapping approach. The manuscript is well-written. The results and figures support the conclusions. I enjoyed reading it. I have several questions and suggestions that I offer below for consideration.

1) On Page 6, line 83, please also consider earlier works that demonstrated multimodal optical mapping of electrophysiology and NADH: see Figure 3 of Lee, et al., 2012: PMID: 22886365 and Figure 5 of Swift, et al., 2008: PMID: 18256457.

2) On Page 6, line 111, please also consider earlier work that used ratiometric optical mapping with motion tracking as a non-contact measurement to demonstrate Katp channel activation in PV-working rabbit hearts: Garrott, et al., 2017: PMID: 28177123.

3) On Page 8, line 164: please consider that mechano-electric feedback is likely to be different between unloaded contracting hearts and loaded PV-working contracting hearts.

4) The two cameras were orthogonally oriented to image the same epicardial region. Please briefly explain how images from the two cameras were aligned to ensure that pixels from each viewpoint imaged the same region of tissue.

5) Figure 3A: please provide the source of the wave shown (RV paced beat) in the figure caption.

6) Figure 4: please briefly explain in the methods section the PSD computation (and frequency resolution) and the parameters used for the computation. Perhaps the Matlab function pwelch was used? Was the computation similar to that of the original articles that computed dominant frequency maps during arrhythmias, ie: Berenfeld, et al., 2000: PMID: 10969749?

7) As shown in Figure 5, it seems that the location chosen for comparing the OAP to the MAP was inconsistent between hearts. Was it not possible to choose a consistent location relative to the MAP tip for OAP analysis?

8) Please also explain why it was not possible to record OAPs *before* placing the MAP electrode, then place the MAP electrode, record the MAP, and compare the OAP at the location of the tip to the recorded MAP. This way the OAP of the tissue beneath the MAP electrode could be generated for the best comparison to the MAP. Was this attempted?

9) Please briefly discuss whether the gentle pressure of the MAP electrode on the epicardial surface could restrict microvascular flow and cause temporary ischemia below the MAP electrode. The pressure of the MAP electrode on the heart is important for generating the signal, yet too much pressure could cause local ischemia, a potential outcome that is rarely discussed in the literature. For example, I would be surprised if the heart shown in Figure 2 of Tse, et al, 2016: PMID: 27135622 was not ischemic. This is important because the OAP is generated from a shallow depth of epicardial tissue but the MAP measures a much larger volume of tissue.

10) Knisley used a glass microelectrode to measure APs for comparison to ratiometric OAPs. Would the authors predict the same outcome as that provided by the MAP if glass microelectrodes were used instead of an MAP electrode?

11) Can the authors explain why bias becomes more negative as mean APD increases, as shown in Figure A1?

12) An additional limitation of the experiments is that non-PV-working heart preparations were studied. The metabolic status of such preparations is lower than that of PV-working preparations, see Wengrowski, et al., PMID: 24337462.

Referee #2:

The manuscript of Kappadan et al., "Combined Emission Ratiometry and Motion Tracking for Optical Mapping of Contracting Hearts: Validation with Monophasic Action Potentials," presents a method of optical mapping in a beating heart without using pharmacological excitation-contraction uncouplers. Several previous reports have presented similar methods, but none are widely used despite the obvious disadvantages of using excitation-contraction uncouplers. The presented method is based on two steps, both contributing to the resolution of motion artifacts. One step is based on ratiometry, which has been known for a quarter of a century but has not been widely used due to the cost of complexity. The other method is motion tracking, which has long been known, but none of the methods are standard in the field.

The proposed method is interesting and is likely to be accepted in the field of optical mapping. This reviewer has several questions:

1. Please provide a photograph and/or 3D rendering of the system. What are the components, and how are they located in 3D space? The design of the system is not entirely clear from the description.

2. Is motion tracking in 3D or 2D? It is important to have 3D tracking, as was shown earlier. 2D projection distorts conduction at the edges. There are several imaging modalities that can track in 3D: ultrasound, structured light, and panoramic imaging.

3. Can you compare the contributions of the two methods to resolving motion artifacts? Which one is more important?

4. It would be helpful to quantify motion artifact, for example, by subtracting the raw signals from the processed data and measuring the difference. Euclidean distance or something similar can be used. This will allow analysis of motion maps.

5. It appears that you excluded edges of the field of view from analysis. Is it because the motion was not resolved at the edges? What was the definition of acceptable motion to include data into analysis?

6 It would be helpful to quantify motion at different degrees of inhibiting contractility. Perhaps this method will be helpful for studying cardiac mechanics.

END OF COMMENTS

Manuscript: Ref: Manuscript ID JP-TFP-2026-291400
Combined Emission Ratiometry and Motion Tracking for Optical Mapping of Contracting Hearts: Validation with Monophasic Action Potentials

We would like to thank the reviewers for their expert opinions, thoughtful commentary, and thorough review of our manuscript. Their detailed feedback has been invaluable in improving the quality and clarity of our work. We have carefully addressed all comments point by point. The corresponding revisions have been highlighted in the revised manuscript. Where applicable, we have also included references to the relevant line numbers in the revised version to facilitate the review of our revisions. We hope that our revisions have sufficiently addressed the editors and reviewers' comments.

REFEREE's COMMENTS:
Referee #1:
Summary
This is an important methods manuscript that provides a robust and relatively comprehensive analysis of non-contact optical mapping of action potentials from the epicardium of contracting unloaded perfused rabbit hearts. Ratiometric motion-tracked optical action potentials are compared to those of a contact measurement, the monophasic action potential, to demonstrate the great utility of this optical mapping approach. The manuscript is well-written. The results and figures support the conclusions. I enjoyed reading it. I have several questions and suggestions that I offer below for consideration.

We thank the reviewer for their positive and constructive comments. We are grateful for their thoughtful suggestions and address each point below.

Specific comments:
1. On Page 6, line 83, please also consider earlier works that demonstrated multimodal optical mapping of electrophysiology and NADH: see Figure 3 of Lee, et al., 2012: PMID: 22886365 and Figure 5 of Swift, et al., 2008: PMID: 18256457.

We thank the reviewer for this helpful suggestion. The studies by *Swift* et al. (2008) and *Lee* et al. (2012) are indeed important early demonstrations of multimodal optical mapping combining electrophysiological measurements with NADH fluorescence imaging, and we agree they provide valuable historical context for our introduction. We have now cited both works at **Page 3, Lines 84-85** of the revised manuscript.

2. On Page 6, line 111, please also consider earlier work that used ratiometric optical mapping with motion tracking as a non-contact measurement to demonstrate Katp channel activation in PV-working rabbit hearts: Garrott, et al., 2017: PMID: 28177123.

We thank the reviewer for this helpful suggestion. *Garrott* et al. (2017) is indeed a highly relevant precedent, demonstrating ratiometric optical mapping combined with motion tracking as a non-contact approach to measure electrophysiological

properties in contracting PV-working rabbit hearts, a preparation with even greater physiological fidelity than our current model. We have now cited this work at **Page 3, Line 113** alongside the other references discussing motion-corrected ratiometric imaging in contracting hearts.

3. On Page 8, line 164: please consider that mechano-electric feedback is likely to be different between unloaded contracting hearts and loaded PV-working contracting hearts.

We thank the reviewer for this important point. We agree that mechano-electric feedback in unloaded contracting hearts is unlikely to fully recapitulate that of loaded, PV-working preparations. As the reviewer's related comments on PV-working versus non-PV-working preparations are addressed in our responses to **comment number 12** below, we have limited the revision here to clarifying in the introduction that our experiments were performed in unloaded, non-PV-working hearts. A more detailed discussion of the differences between these preparations have been added to the Limitations and Future (**Page 17, Lines 668-674**).

Revised text (**Page 5, Lines 165-167**):
"*Motion-corrected optical action potentials are quantitatively validated against simultaneously recorded MAPs, enabling rigorous assessment of action potential repolarization timing across multiple repolarization levels in contracting hearts that preserve excitation-contraction coupling and elements of mechano-electric feedback, albeit without the full physiological preload and afterload conditions of pressure-volume (PV) working heart preparations.*"

4. The two cameras were orthogonally oriented to image the same epicardial region. Please briefly explain how images from the two cameras were aligned to ensure that pixels from each viewpoint imaged the same region of tissue.

We thank the reviewer for this important point and agree that the alignment procedure warrants explicit description. Camera alignment was performed in two stages. In the first, mechanical stage, a projected point light source was introduced into the optical system and the camera fitted with the >620 nm filter was held fixed as the reference. The second camera was then adjusted through rigid-body transformations (translation and rotation) until the projected point appeared at the same pixel coordinates in both fields of view. In the second, verification stage, shared anatomical landmarks visible in both emission channels - most notably epicardial vessels - were used to confirm that matched pixels corresponded to the same tissue location.

The added text (**Page 6, Lines 203-212**) reads:
"*Camera alignment was achieved using a two-step procedure. First, a projected point light source was introduced into the optical system for gross mechanical co-alignment. The camera fitted with the >620nm filter was held fixed as the reference, while the second camera was adjusted through purely rigid-body transformations until the projected light source appeared at the same position in both fields of view. Second, computational verification of the registration was performed by confirming*

the correspondence of shared anatomical landmarks visible in both channels, such as epicardial vessels, ensuring that matched pixels represented the same tissue location."

5. Figure 3A: please provide the source of the wave shown (RV paced beat) in the figure caption.

We thank the reviewer for this helpful comment. The representative propagation sequence shown in **Figure 3A** corresponds to a paced beat delivered from the mid-right ventricle, located on the posterior side of the imaged epicardial surface. We have revised the **Figure 3A** caption (**Page 26**, **Lines 924–925**) to include this information.

The revised caption reads:
" *Representative wave propagation snapshots obtained from optical mapping videos during a right ventricular (RV) paced beat, with pacing delivered from the mid-RV region located on the posterior side of the imaged surface. (a) Wave propagation before motion correction, where motion artefacts appear as high-frequency network-like spatial patterns and spurious excitation signals superimposed on and distorting the action potential wave pattern. (b) Wave propagation after motion correction, with marked reduction of motion artefacts and clearer wavefront structure.*"

6. Figure 4: please briefly explain in the methods section the PSD computation (and frequency resolution) and the parameters used for the computation. Perhaps the Matlab function pwelch was used? Was the computation similar to that of the original articles that computed dominant frequency maps during arrhythmias, ie: Berenfeld, et al., 2000: PMID: 10969749?

We thank the reviewer for this important comment. In the original manuscript, dominant frequency analysis was performed using a direct Fourier transform of each signal rather than a Welch-based power spectral density estimate. We agree that Welch's method is preferable for this application: by averaging modified periodograms over overlapping segments, it reduces the variance of the spectral estimate and yields a more stable identification of the dominant peak, particularly for relatively short and noisy biological recordings. Our approach is now consistent with that established by *Berenfeld* et al. (2000) and subsequent dominant-frequency mapping studies. We have recalculated the PSD using Welch's method and revised the manuscript accordingly. Signals were mean-centered and analyzed in MATLAB using pwelch with a Hann window, 512-sample segments, 50% overlap, and a 512-point FFT at a sampling frequency of 250 Hz, giving a frequency resolution of 0.488 Hz - comparable to the 0.47 Hz resolution reported by *Berenfeld* et al. (2000).

Revised Methods (**Page 9, Lines 317-322**):
"*For ventricular fibrillation recordings, dominant frequency maps were computed using Welch power spectral density estimation. Power spectral density was estimated using a Hann window, 512-sample segments, 50% overlap, and a 512-point fast Fourier transform at a sampling frequency of 250 Hz, giving a frequency resolution of 0.488 Hz. Dominant frequency was defined as the frequency with the highest spectral power.*"

Revised Figure 4 caption (**Page 27, Lines 946-950**):
"*Figure 4. Dominant frequency of ventricular fibrillation before and after motion correction.*
(A) Ventricular fibrillation recordings before motion correction. (a) Representative ventricular fibrillation (VF) time traces. (b) Corresponding normalised Welch-derived power spectra. (c) Dominant frequency map overlaid on a representative single-frame heart image.
(B) Ventricular fibrillation recordings after motion correction. (a) VF time traces. (b) Corresponding normalised Welch-derived power spectra. (c) Dominant frequency map after correction."

The updated Figure 4 is shown below:

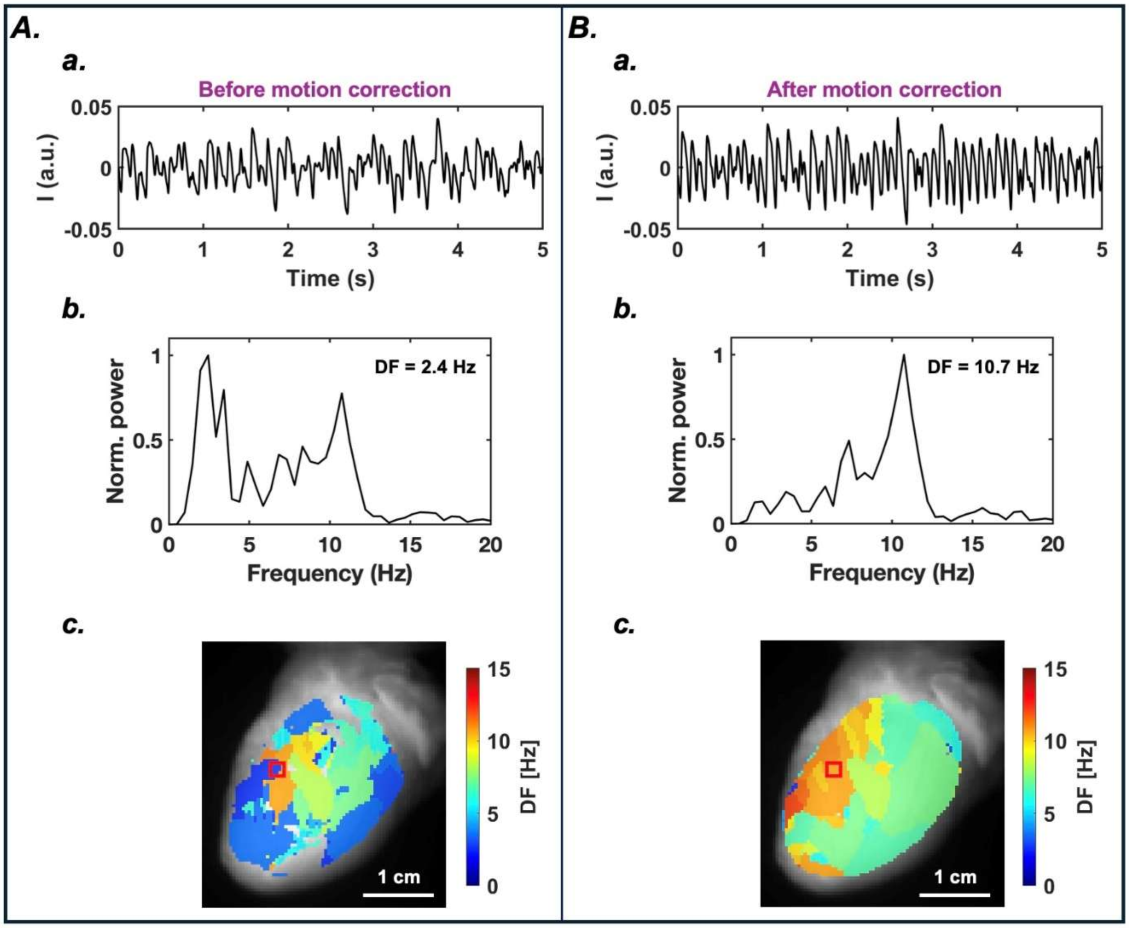

7. As shown in Figure 5, it seems that the location chosen for comparing the OAP to the MAP was inconsistent between hearts. Was it not possible to choose a consistent location relative to the MAP tip for OAP analysis?

We thank the reviewer for this important point. We agree that, ideally, OAPs would be sampled from the same position relative to the MAP electrode tip in every heart. However, this was not always feasible in practice because the usable optical sampling region differed between preparations. Variations in MAP electrode

placement, local occlusion or shadowing by the electrode, and the proximity of the recording site to the edge of the field of view meant that applying a fixed relative offset would, in some hearts, have forced analysis into regions with poorer optical signal quality. We therefore adopted a consistent practical approach: in each heart, OAPs were sampled from pixels as close as possible to the MAP electrode tip while avoiding pixels directly beneath the electrode or in visibly occluded or shadowed regions. This was particularly important near the edges of the heart, where reduced tissue visibility, out-of-plane motion, and more difficult motion tracking on the curved epicardial surface often made optical measurements less reliable. Thus, we prioritized local signal quality and anatomical proximity to the MAP site over strict geometric matching when these were in conflict.

The MAP electrode placement was intentionally varied across hearts to demonstrate that the motion-correction framework performed robustly across different ventricular regions, rather than only at a single fixed site. Although this introduced some variability in the relative OAP-MAP sampling geometry, the analysed optical pixels in all hearts remained immediately adjacent to the MAP recording site. The strong agreement between OAP- and MAP-derived repolarization measurements across hearts and repolarization levels supports the robustness of this comparison.

To further illustrate this point, we have included an additional example showing two hearts in which approximately matched OAP sampling positions relative to the MAP tip were achievable. As noted above, however, this level of consistency could not be maintained in every preparation because of the experimental constraints described.

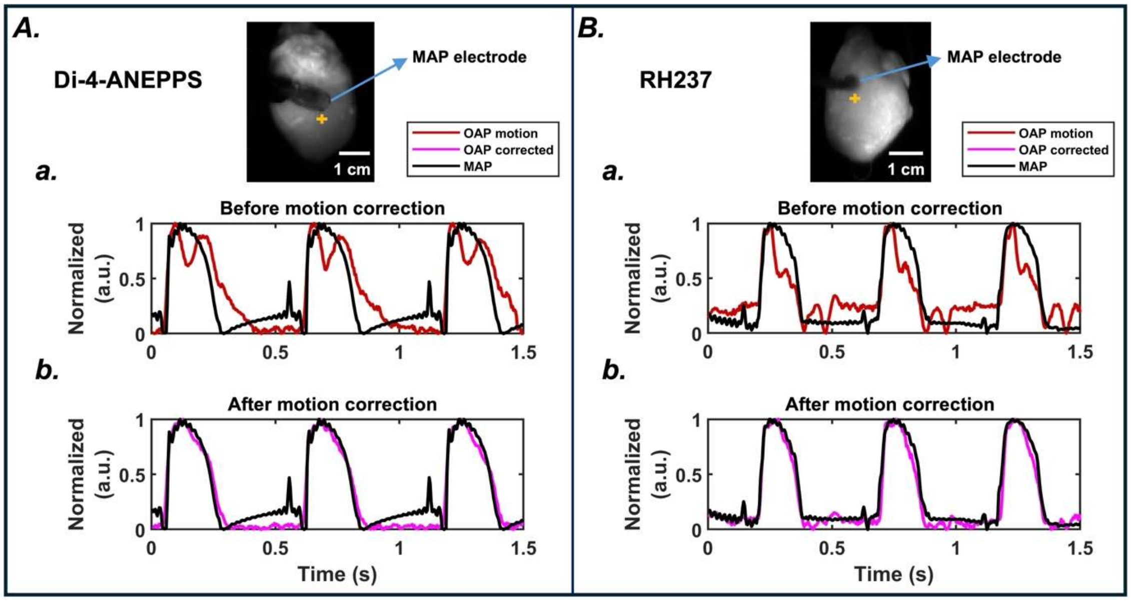

Figure R1. Representative OAPs and MAPs sampled at approximately matched locations relative to the MAP electrode tip in two different hearts.

Heart images (top) show the MAP electrode position and the OAP sampling pixel (yellow cross) for (**A**) a Di-4-ANEPPS-labelled heart and (**B**) an RH237-labelled heart. For each heart, representative traces are shown (a) before motion correction and (b) after motion correction, comparing the uncorrected OAP (red), motion-corrected OAP (magenta), and simultaneously recorded MAP (black).

In both preparations, the OAP sampling region was placed on the anterior epicardial surface at a comparable distance from the MAP electrode tip, and motion-corrected OAPs closely track the MAP waveform across successive beats.

8. Please also explain why it was not possible to record OAPs *before* placing the MAP electrode, then place the MAP electrode, record the MAP, and compare the OAP at the location of the tip to the recorded MAP. This way the OAP of the tissue beneath the MAP electrode could be generated for the best comparison to the MAP. Was this attempted?

We thank the reviewer for this thoughtful suggestion. We did attempt this approach in a small number of hearts, with encouraging results in some cases (attached below), but the number of successful recordings was too limited to support formal statistical analysis. We ultimately adopted the ***simultaneous*** (*rather than sequential*) MAP–OAP approach because it proved more robust and reproducible across preparations, for several reasons. First, the mechanical conditions of the preparation often changed once the MAP electrode was applied, so motion patterns before and during electrode contact were not necessarily equivalent, and pre-contact OAPs did not always reflect the optical conditions present during MAP acquisition. Second, MAP recording depends on firm, stable tissue apposition; acquiring an OAP recording first and then re-establishing good MAP contact under dark-room conditions was experimentally difficult and poorly reproducible. Third, the time required to achieve satisfactory MAP contact after OAP recording could introduce physiological drift, which was non-negligible in our experiments. Fourth, MAP contact may alter local tissue conditions such as focal ischaemia, so optical signals recorded prior to electrode placement cannot be assumed identical to those recorded during MAP acquisition. Finally, precise tracking of the MAP tip position is difficult in optical images acquired before the electrode is in place. For these reasons, although the sequential strategy was explored, the ***simultaneous*** approach proved more practical and reliable for systematic comparison across hearts.

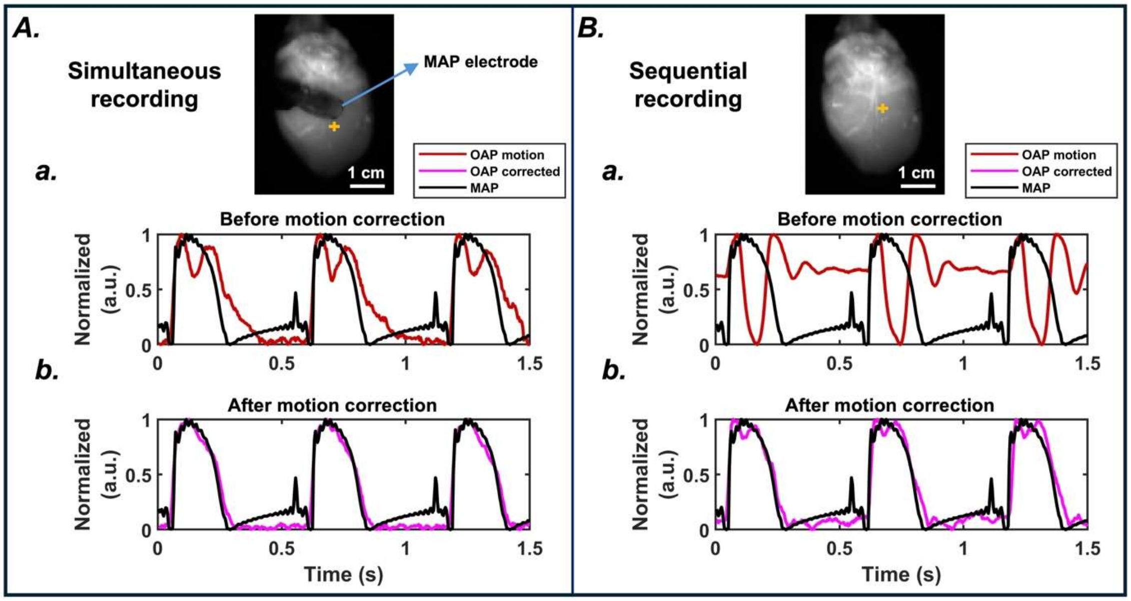

Figure R2: Representative OAPs and MAPs recorded from a rabbit heart during sinus rhythm using Di-4-ANEPPS, obtained under simultaneous and sequential recording conditions.

(A) Overlay of OAP and MAP traces before (a) and after (b) motion correction during simultaneous recording. Optical signals were extracted from pixels located near the MAP electrode. Prior to motion correction, OAPs exhibit substantial distortion and deviate markedly from the MAPs. After correction, OAPs show reduced distortion and improved agreement with MAPs in both waveform morphology and timing.

(B) Overlay of OAP and MAP traces before (a) and after (b) motion correction during sequential recording (MAP followed by OAP). MAP and OAP were separated by approximately15 seconds. Optical signals were extracted from pixels corresponding to the former MAP electrode location. Following motion correction, OAPs display clearer morphology and closer temporal correspondence with MAP signals.

A representative optical frame acquired during simultaneous recording (with the MAP electrode in place) and during sequential recording (after MAP electrode removal) is shown at the top of panels A and B, respectively. The optical recording site is indicated by an orange plus symbol (+).

9. Please briefly discuss whether the gentle pressure of the MAP electrode on the epicardial surface could restrict microvascular flow and cause temporary ischemia below the MAP electrode. The pressure of the MAP electrode on the heart is important for generating the signal, yet too much pressure could cause local ischemia, a potential outcome that is rarely discussed in the literature. For example, I would be surprised if the heart shown in Figure 2 of Tse, et al, 2016: PMID: 27135622 was not ischemic. This is important because

the OAP is generated from a shallow depth of epicardial tissue but the MAP measures a much larger volume of tissue.

We thank the reviewer for this important point. We agree that MAP recordings have inherent methodological limitations and may not constitute a perfect reference standard. In particular, MAP acquisition requires firm contact between the electrode and the epicardial surface, and excessive local pressure could in principle alter local mechanical conditions or restrict superficial microvascular perfusion, thereby introducing regional ischaemia beneath the electrode. In addition, MAP and OAP do not sample identical tissue volumes: optical signals arise predominantly from a relatively superficial epicardial layer, whereas MAP recordings reflect electrical activity over a larger local tissue volume. We have now acknowledged these issues explicitly in the Limitations section of the revised manuscript.

Page 17, Lines 641-651: "*In addition, some mismatch may reflect limitations inherent to MAP recording itself, which requires stable, firm contact between the electrode and the epicardial surface. Although this contact is necessary to obtain a MAP signal, excessive local pressure could theoretically alter local mechanical conditions, restrict superficial microvascular perfusion, and thereby produce regional ischaemic effects beneath the electrode. Moreover, optical action potentials and MAPs do not sample identical tissue volumes: optical recordings predominantly reflect a relatively shallow epicardial layer, whereas MAP recordings reflect electrical activity arising from a larger local myocardial volume under and around the contact site. These factors may have contributed to the small differences observed between MAPs and nearby optical action potentials.*"

10. Knisley used a glass microelectrode to measure APs for comparison to ratiometric OAPs. Would the authors predict the same outcome as that provided by the MAP if glass microelectrodes were used instead of an MAP electrode?

We thank the reviewer for this insightful question. We agree that a glass microelectrode would provide a different reference measurement from a MAP electrode and would avoid certain MAP-specific limitations related to epicardial contact pressure. However, we would not necessarily expect identical agreement between OAPs and glass microelectrode recordings. Glass microelectrodes measure transmembrane potential at a highly localized site, whereas OAPs represent an optically weighted signal arising from a superficial tissue volume. Conversely, MAP recordings provide a more integrated local extracellular surrogate of action potential morphology but are influenced by electrode contact conditions. We therefore expect that the overall electrophysiological trends, particularly repolarization timing, would likely remain similar, but the quantitative agreement might differ depending on the exact recording location, stability of impalement, and local tissue heterogeneity.

11. Can the authors explain why bias becomes more negative as mean APD increases, as shown in Figure A1?

We thank the reviewer for this observation. Although a slight visual tendency toward

more negative differences at larger mean APD values is apparent, the magnitude of the bias decreased substantially across repolarization levels, from a modest offset at APD_{50} (6.14 ms) to very small offsets at APD_{70} (1.3 ms) and APD_{80} (-0.7 ms). We therefore interpret the APD_{70} and APD_{80} findings as showing minimal residual disagreement between modalities rather than evidence of inadequate motion correction. At these later repolarization levels, the remaining offsets are very small and are more likely to reflect the expected minor mismatch between two different recording modalities, together with the slight spatial separation between the MAP electrode and the neighbouring optical recording site used for comparison. In contrast, the somewhat larger bias at APD_{50} may indicate greater residual sensitivity during earlier repolarization, when mechanical deformation is more pronounced, as already noted in the manuscript at **Line 556**. This may reflect limitations of the present 2D motion-correction framework, which does not explicitly account for out-of-plane motion or full three-dimensional tissue deformation, as discussed in the limitations section. This distinction is also biologically relevant, because later repolarization metrics such as APD_{70} and APD_{80} are more closely related to recovery of excitability and refractoriness than APD_{50}, and heterogeneity in repolarization/refractoriness is closely linked to arrhythmogenesis.

12. An additional limitation of the experiments is that non-PV-working heart preparations were studied. The metabolic status of such preparations is lower than that of PV-working preparations, see Wengrowski, et al., PMID: 24337462.

We thank the reviewer for this important point. We agree that the non-PV-working Langendorff preparation used in this study does not reproduce the loading conditions or metabolic demand of a PV-working heart. We have now revised the Discussion to explicitly acknowledge that this preparation lacks pressure–volume work, is therefore likely to operate at a lower metabolic demand, and may differ from PV-working preparations in both metabolic state and mechano-electric feedback as mentioned previously in point 3. This limitation has now been added to the manuscript.

Page 17, Lines 668-674: "*Fourth, experiments were performed in unloaded, non-PV-working Langendorff-perfused hearts. This preparation does not reproduce the pressure-volume work ofthe intact heart and is likely to operate at a lower metabolic demand than PV-working preparations (Wengrowski et al., 2014). As metabolic state and mechanical loading both influence cardiac electrophysiology, including mechano-electric feedback, extension of the present framework to PV-working preparations represents an important direction for future work.*"

Referee #2:

Summary

The manuscript of Kappadan et al., "Combined Emission Ratiometry and Motion Tracking for Optical Mapping of Contracting Hearts: Validation with Monophasic Action Potentials," presents a method of optical mapping in a beating heart without using pharmacological excitation-contraction uncouplers. Several previous reports have presented similar methods, but none are widely used despite the obvious disadvantages of using excitation-contraction uncouplers. The presented method is based on two steps, both contributing to the resolution of motion artifacts. One step is based on

ratiometry, which has been known for a quarter of a century but has not been widely used due to the cost of complexity. The other method is motion tracking, which has long been known, but none of the methods are standard in the field.

The proposed method is interesting and is likely to be accepted in the field of optical mapping. This reviewer has several questions.

We thank the reviewer for their positive and constructive comments. We are grateful for their thoughtful suggestions and address each point below.

Specific comments:
1. Please provide a photograph and/or 3D rendering of the system. What are the components, and how are they located in 3D space? The design of the system is not entirely clear from the description.

We thank the reviewer for this helpful comment and agree that the original description did not make the system configuration sufficiently clear. We would like to clarify that our setup is not a 3D imaging or 3D rendering system. Rather, it is based on a conventional voltage-calcium dual optical mapping configuration, in which the optical path is separated using dichroic optics and emission filters to direct the respective signals to different cameras. This general type of arrangement is well established in cardiac optical mapping systems. The distinguishing feature of our system is therefore not a 3D architecture, but that we modified the dichroic mirror and camera filters so that the setup was compatible with the emission spectra required for ratiometric imaging. To make this clearer for readers, we have now added a photo of the experimental system in the Appendix to help clarify it.

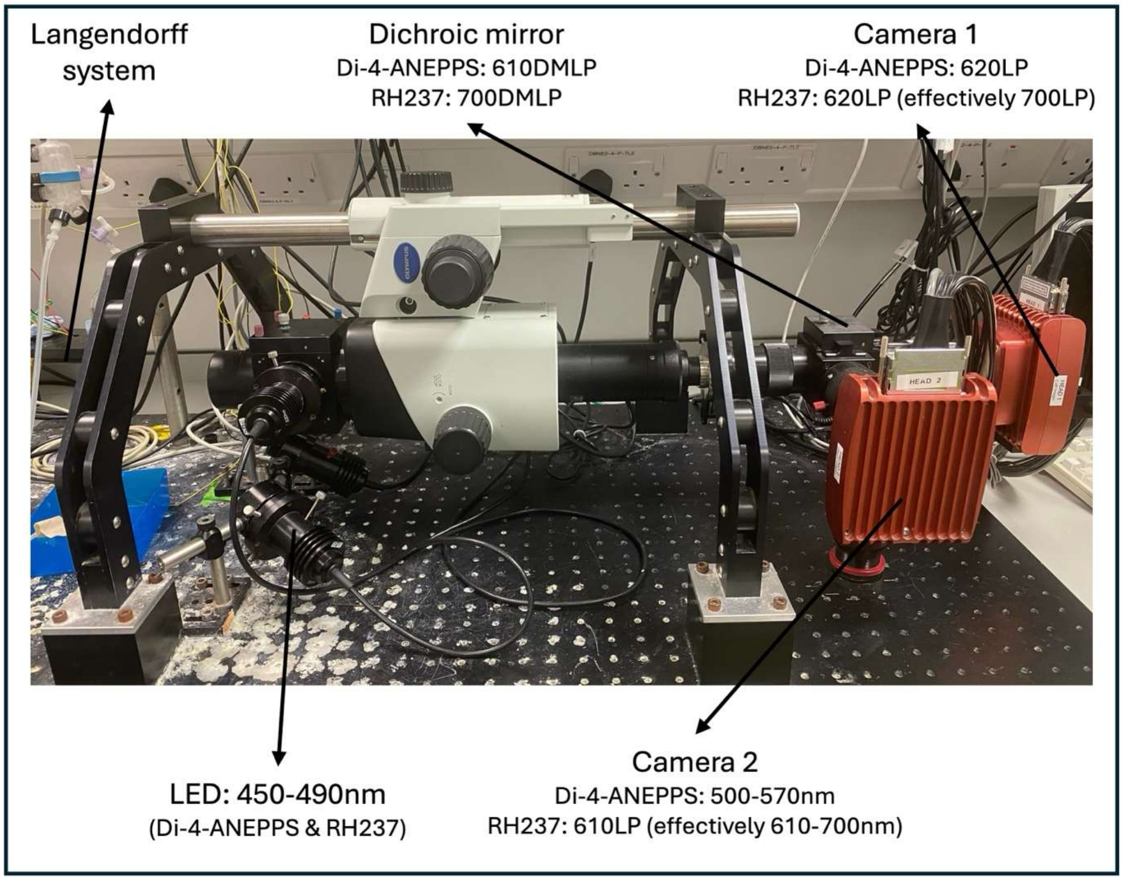

"***Figure A1. Experimental setup for ratiometric optical mapping of cardiac action potentials.***
A Langendorff-perfused heart is illuminated by blue LEDs (450-490 nm excitation) used to excite both voltage-sensitive dyes, Di-4-ANEPPS and RH237. Emitted fluorescence is split by a dichroic mirror (610DMLP for Di-4-ANEPPS; 700DMLP for RH237) and directed to two orthogonally oriented CMOS cameras (128 × 80 pixels, 250 Hz) aligned to image the same region of the epicardium. Camera 1 is fitted with a 620LP filter (effectively 700LP for RH237), capturing the long-wavelength emission channel (red for Di-4-ANEPPS; near-infrared for RH237). Camera 2 is fitted with a 535 ± 35 nm filter for Di-4-ANEPPS (green channel) or a 610LP filter (effectively 610–700 nm) for RH237 (red channel), capturing the short-wavelength emission. This dual-camera configuration enables simultaneous acquisition of the two spectral components required for ratiometric imaging of transmembrane potential, reducing motion and illumination artefacts." (**Page 32, Lines 1019-1032**)

2. Is motion tracking in 3D or 2D? It is important to have 3D tracking, as was shown earlier. 2D projection distorts conduction at the edges. There are several imaging modalities that can track in 3D: ultrasound, structured light, and panoramic imaging.

We thank the reviewer for this important point. In the present study, motion tracking was performed using the Farnebäck method, a dense 2D optical-flow algorithm that estimates frame-to-frame in-plane displacement from image intensity patterns. As

such, our implementation is inherently 2D rather than 3D. We agree that this is an important limitation, because out-of-plane motion and surface curvature cannot be fully captured by a 2D approach, particularly when deformation is large or toward the edges of the field of view, where projection of the curved epicardial surface onto a 2D image plane may distort conduction measurements which was already acknowledged in the limitation section.

We also agree that 3D motion tracking and panoramic approaches would provide a more complete solution, both by improving motion tracking itself and by enabling electrophysiological signals to be mapped onto the true 3D cardiac surface. Techniques such as structured light, ultrasound-based approaches, and panoramic imaging are therefore highly relevant and represent an important direction for future development.

Nevertheless, the aim of the present work was to evaluate whether a comparatively simple and accessible 2D optical-flow-based correction could still mitigate motion artefacts and recover physiologically interpretable signals in contracting-heart optical mapping. Our results suggest that, although incomplete, this approach can still be useful in some settings.

We have now explicitly clarified this limitation in the limitations section on **Pages 16-17**, **Lines 622-634**: "*This limitation is particularly relevant near the edges of the field of view, where the curved epicardial surface is viewed obliquely and where 2D projection may distort apparent activation patterns, conduction direction, or conduction velocity. Accordingly, conduction and repolarization patterns in peripheral regions should be interpreted cautiously, and quantitative analyses in the present study were focused on regions where corrected signals remained physiologically plausible and free from obvious residual distortion. Future integration with panoramic or multi-view optical mapping systems may help address this limitation by enabling more comprehensive characterization of 3D cardiac structural and mechanical dynamics. Other 3D motion-tracking approaches, including structured-light surface imaging or ultrasound-based motion assessment, may also provide complementary strategies for capturing out-of-plane deformation in future studies.*"

3. Can you compare the contributions of the two methods to resolving motion artifacts? Which one is more important?

We thank the reviewer for this important point. In our dataset, the relative contributions of the two correction methods can be estimated from the Bland-Altman analysis of APD_{80} measured from raw contracting signals, motion tracking only, ratiometry only, and the combined approach. Both methods improved agreement relative to raw contracting signals, but ratiometry alone produced a larger reduction in systematic bias (from 26.40 ms to 12.21 ms) than motion tracking alone (from 26.40 ms to 20.91 ms). This suggests that, for APD_{80} in our preparation, ratiometry made the larger individual contribution. However, neither method alone was sufficient, as both retained substantial residual bias. The best performance was achieved when motion tracking and ratiometry were combined, reducing the bias further to -0.70 ms. We therefore interpret the two methods as complementary rather than interchangeable: ratiometry mainly suppresses shared photometric/intensity

fluctuations, whereas motion tracking corrects spatial misregistration caused by tissue displacement.

We have revised the manuscript to make this discussion clearer (**Page 14**, **Line 534-545**): "*When applied individually, each method mitigates only part of the motion-related distortion. In the present dataset, this complementarity was also evident quantitatively from the Bland-Altman analysis of APD80. Relative to raw contracting optical signals, both correction methods improved agreement with MAP recordings, but ratiometry alone produced a larger reduction in systematic bias than motion tracking alone, reducing the mean bias from 26.40 ms to 12.21 ms compared with 20.91 ms after motion tracking alone. This suggests that, for APD80 in this preparation, ratiometry made the larger individual contribution to reducing systematic APD error. However, neither method alone eliminated the residual bias, whereas the combined approach reduced the mean bias further to -0.70 ms. These findings support the interpretation that the two approaches are complementary rather than interchangeable.*"

4. It would be helpful to quantify motion artifact, for example, by subtracting the raw signals from the processed data and measuring the difference. Euclidean distance or something similar can be used. This will allow analysis of motion maps.

We thank the reviewer for this suggestion. We agree that quantitative assessment of correction effects would be valuable. However, subtracting raw and processed optical signals would primarily quantify the magnitude of signal alteration introduced by the correction pipeline, rather than provide a direct measure of motion artefact or tissue motion itself. In our setting, the corrected signals reflect the combined effects of 2D motion tracking, ratiometric processing, interpolation/warping, and residual artefact. Importantly, the amount of residual artefact remaining after correction is unknown and may vary spatially and temporally, making any such difference map difficult to interpret biologically. As a result, the resulting signal differences cannot be interpreted as a validated motion map or as a measurement of cardiac mechanics. More generally, our Farnebäck-based approach estimates apparent image-plane displacement rather than true 3D myocardial motion, and optical-flow estimates may be affected by out-of-plane motion, large deformation, and intensity changes unrelated to geometric displacement. We therefore consider this framework to be a motion-compensation approach for improving electrophysiological signal interpretation, rather than a method for quantifying myocardial mechanics. Proper assessment of cardiac motion/mechanics would require dedicated and ideally validated 3D motion measurements, and would certainly form the basis of another interesting study.

5. It appears that you excluded edges of the field of view from analysis. Is it because the motion was not resolved at the edges? What was the definition of acceptable motion to include data into analysis?

We thank the reviewer for this comment. Peripheral regions of the field of view were excluded because motion estimation is less reliable near image boundaries. The Farnebäck optical-flow algorithm estimates displacement from local image

neighbourhoods using polynomial expansion; at the myocardium–background boundary, the local support region may contain both myocardial and non-myocardial pixels, reducing tracking robustness. Peripheral regions are also more susceptible to boundary effects, limited local image features, and complex out-of-plane deformation. We therefore applied a conservative inward exclusion of approximately 8–12 pixels from the visible myocardial boundary, corresponding to approximately 10–15% of the heart width in our 128 × 80 pixel recordings, to ensure that the local neighbourhood used for motion estimation remained predominantly within myocardial tissue.

Following this exclusion, corrected signals were visually reviewed across multiple regions of the heart before APD calculation. Regions were retained only when corrected optical action potentials remained physiologically plausible and APD maps showed no obvious residual localised distortion or artifactual structure. We agree that more objective pixel-wise confidence measures or motion-quality metrics would be valuable in future work, particularly in diseased preparations with greater intrinsic electrophysiological heterogeneity, where this pragmatic quality-control approach may be less reliable.

We have added this as an additional limitation in the manuscript (**Page 17–18**, **Lines 676–697**): " *Finally, a limitation of the present study is the absence of a simple, objective, one-step method for defining which regions are sufficiently reliable for quantitative electrophysiological analysis after motion correction. Although the combination of motion tracking and ratiometry substantially reduced motion artefact, the final selection of analysable regions still required several pragmatic quality-control steps, including conservative exclusion of peripheral regions, visual review of corrected signals across multiple myocardial regions, and exclusion of areas where APD calculation was unreliable or physiologically implausible. This reflects a broader technical challenge in contracting-heart optical mapping, where even small residual motion can distort optical action potentials and calcium transients, and where motion correction remains difficult in the presence of boundary effects, out-of-plane deformation, and spatially heterogeneous tissue motion. In the present study, this pragmatic approach was considered appropriate because the preparations were predominantly healthy hearts, in which large pathological APD and mechanical heterogeneity were not expected. However, the approach remains partly operator-dependent and may introduce greater uncertainty in diseased preparations, where true regional electrophysiological and mechanical heterogeneity may be more pronounced and therefore harder to distinguish from residual tracking uncertainty or motion-related artefact. Future studies would benefit from objective pixel-wise motion-quality metrics, confidence maps, or automated criteria that integrate tracking reliability, residual motion artefact, and signal or APD quality into a reproducible assessment of whether each region is suitable for quantitative analysis.*"

6. It would be helpful to quantify motion at different degrees of inhibiting contractility. Perhaps this method will be helpful for studying cardiac mechanics.

We thank the reviewer for this nice suggestion. We agree that quantifying motion across different degrees of contractility inhibition is an interesting direction. However,

the present Farnebäck-based pipeline was developed as a 2D image-registration approach to reduce motion artefact in optical mapping signals, rather than as a validated method for measuring true myocardial motion or mechanics. Optical-flow methods estimate apparent image-plane displacement under assumptions such as brightness constancy and spatial regularity, and their performance can degrade with out-of-plane motion, large deformation, occlusion, and photometric changes. Therefore, although the algorithm can provide a dense 2D tracking field for signal stabilization, we do not consider this equivalent to a true motion map or a quantitative assessment of cardiac mechanics. We do however agree that this concept could be interesting to test in another study.

Dear Professor Ng,

Re: JP-TFP-2026-291400R1 "Combined Emission Ratiometry and Motion Tracking for Optical Mapping of Contracting Hearts: Validation with Monophasic Action Potentials" by Vineesh Kappadan, Zhen Hua, Johanna B Tonko, Najmah Mohamed, Jan Lebert, Paraskevas Efstathiou, Yasser Abdelghani, Danya Agha-Jaffar, Anies Sohi, Xianbo Sun, Jean-Baptiste Guichard, Jan Christoph, Nicholas S Peters, and Fu Siong Ng

We are pleased to tell you that your paper has been accepted for publication in The Journal of Physiology.

TRANSPARENT PEER REVIEW POLICY: To improve the transparency of its peer review process The Journal of Physiology publishes online, as supporting information, the peer review history of all articles accepted for publication. Readers will have access to decision letters, including Editors' comments and referee reports, for each version of the manuscript, as well as any author responses to peer review comments. Referees can decide whether or not they wish to be named on the peer review history document.

The last Word (or similar) version of the manuscript provided will be used by the Production Editor to prepare your proof. When this is ready you will receive an email containing a link to Wiley's Online Proofing System. The proof should be thoroughly checked and corrected as promptly as possible.

Authors should note that it is too late at this point to offer corrections prior to proofing. Major corrections at proof stage, such as changes to figures, will be referred to the Editors for approval before they can be incorporated. Only minor changes, such as to style and consistency, should be made at proof stage. Changes that need to be made after proof stage will usually require a formal correction notice.

All queries at proof stage should be sent to: TJP@wiley.com

Are you on Twitter? Once your paper is online, why not share your achievement with your followers? Please tag The Journal (@jphysiol) in any tweets and we will share your accepted paper with our 30,000 followers!

If you would like to receive our 'Research Roundup', a monthly newsletter highlighting the cutting-edge research published in The Physiological Society's family of journals (The Journal of Physiology, Experimental Physiology and Physiological Reports), please click this link, fill in your name and email address and select 'Research Roundup':
https://www.physoc.org/journals-and-media/membernews/

Yours sincerely,

Eleonora Grandi
Senior Editor
The Journal of Physiology

P.S. - You can help your research get the attention it deserves! Check out Wiley's free Promotion Guide for best-practice recommendations for promoting your work at www.wileyauthors.com/eeo/guide. You can learn more about Wiley Editing Services which offers professional video, design, and writing services to create shareable video abstracts, infographics, conference posters, lay summaries, and research news stories for your research at www.wileyauthors.com/eeo/promotion.

• IMPORTANT NOTICE ABOUT OPEN ACCESS: To assist authors whose funding agencies mandate immediate public access to published research findings, The Journal of Physiology allows authors to pay an Open Access (OA) fee to have their papers made freely available immediately on publication.

The Corresponding Author will receive an email from Wiley with details on how to register or log in to Wiley Authors where you will be able to place an order.

You can check if your funder or institution has a Wiley Open Access Account here:
https://authors.wiley.com/author-resources/Journal-Authors/open-access/author-compliance-tool.html

----

EDITOR COMMENTS

Reviewing Editor:

Thank you for addressing the reviewers' concerns in your revised paper.

Senior Editor:

Congratulations!!

REFEREE COMMENTS:

Referee #1:

Thank you for considering my questions and suggestions. The revisions were nicely done and the manuscript has improved. This work will be an important resource for EP scientists and engineers who use optical mapping to study excised perfused hearts.

Referee #2:

Thank you for your excellent revisions.

****
